# Hall current and morphological effects on MHD micropolar non-Newtonian tri-hybrid nanofluid flow between two parallel surfaces

**DOI:** 10.1038/s41598-022-19625-3

**Published:** 2022-10-05

**Authors:** Abdul Rauf, Faisal, Nehad Ali Shah, Thongchai Botmart

**Affiliations:** 1 Department of Mathematics, Air University Multan Campus, Chak 5-Faiz, Bahawalpur Road, Multan, Pakistan; 2 Department of Mechanical Engineering, Sejong University, Seoul, 05006 South Korea; 3 Department of Mathematics, Faculty of Science, Khon Kaen University, Khon Kaen, 40002 Thailand

**Keywords:** Mathematics and computing, Nanoscience and technology

## Abstract

In the present work, the magnetohydrodynamic flow and heat transfer of a micropolar tri-hybrid nanofluid between two porous surfaces inside a rotating system has been examined. A tri-hybrid nanofluid is a new idea in the research area, which gives a better heat transfer rate as compared to hybrid nanofluid and nanofluid. We also incorporated the thermal radiation effects and Hall current in this article. The similarity techniques are used to reduce the governing nonlinear PDEs to a set of ODEs. For the numerical solution of the considered problem, we have used the MATLAB-based Bvp4c method. The results are presented for tri-hybrid Fe_3_O_4_-Al_2_O_3_-TiO_2_/H_2_O nanofluid. The main focus of this study is to examine the magnetohydrodynamic heat transfer and tri-hybrid nanofluid flow in a rotating system between two orthogonal permeable plates by taking into account the Hall current and thermal radiation effects. The obtained results have been explained with the help of graphical illustrations and tables. It is observed that the heat transfer rate of tri-hybrid nanofluid is greater than as compared to hybrid nanofluid and nanofluid. The increasing behavior is also noticed in micro rotational velocity for augmented values of ${R}_{0}$, $Ha,$ and $\beta$. The larger values of ${\phi }_{1}$, ${\phi }_{2}$, and ${\phi }_{3}$ result in the decrement of SFC and increment in Nusselt number in both (suction and injection) cases.

## Introduction

Many natural fluids accumulate small TC (thermal conductivity) for heat transfer, which is regarded as a significant barrier in the development of the thermal flow system. The manufacturing of numerous devices and components used in industrial and technological applications has advanced significantly in the modern world. For example, in the industry, several gadgets due to the resistance of electricity begin to increase their temperature with time. Because of the electric resistance, the heat-carrying capability of such gadgets was reduced, resulting in a technical fault. Heat intemperance from various devices and components is required to reduce the risk of a technical fault. As a result, industrialists utilize fluids like water, air, and lubricants to manage proper heat transport. However, at the industrial level, such natural fluids do not meet the requirements. To accomplish these objectives, investigators have investigated various ways to keep fluid flow and transmission of heat within the design limit. One of these ways is to add nanoparticles (NPs) to various natural fluids because of their lower TC. Furthermore, the properties of nanofluids can be designed for a particular application if required. The idea of the addition of NPs was introduced by Choi and Eastman^1^. Ellahi et al.^2^ studied the flow of nanoparticles over a wedge in a permeable medium using a mixed convective fluid flow. They also included the effects of various particle morphologies in this research. By considering Brownian motion, Dogonchi and Ganji^3^ investigated electrically conductive heat transfer for buoyancy and nanofluid flow across a rigid surface. They have identified the increase in temperature and velocity profiles for growth in radiation parameters in this investigation. They also discovered in this investigation that as the magnetic parameter rises, the skin friction coefficients increase. Considering Joule's heat effects, Dogonchi and Ganji^4^ investigated the MHD (magnetohydrodynamic) flow of a nanofluid and heat transfer between two surfaces. With the enhancement in the magnitude of the Schmidt number, they observed enhancement in the concentration, Nusselt number, and temperature profiles in this investigation. Using a cylindrically shaped annulus, Oudina and Bessah^5^ examined the convection of heat transmission for nanoparticles is numerically modeled. Gourari et al.^6^ statistically investigated the natural convective flow between coaxial inclined cylinders. Using several base fluids, Oudina^7^ investigated convective heat transfer for the titania nanofluid. By examining the effects of various forms, Raza et al.^8^ investigated the MHD flow for molybdenum disulfide NPs inside a conduit. They investigated the influence of NPs on the MHD flow numerically in this study. In this investigation, they discovered that increasing the volume fraction leads to an increase in the Nusselt number. Furthermore, they found in this study that for increased values of wall expansion ratio, the velocity profile boosted from the bottom wall to the Centre of the channel, then fell.

The flow behavior of a moving conducting liquid is described by magnetohydrodynamics (MHD), which polarizes it. Magnetic field effects are studied in industrial operations such as fuel manufacturing, nuclear power plants, crystal fabrication, electrical generators, and aerodynamics, among others. The field of MHD was presented by Alfven et al.^9^. Emad et al.^10^ presented a numerical treatment of a hybrid magnetic nanomaterial in a porous stretching medium. Chamkha et al.^11^ studied the natural convection of a magnetohydrodynamic nanofluid in an insertion under the influence of thermal radiation using the control volume-based finite element approach, as well as the form factor of nanoparticles Using the Duan Rach Approach, for turbine cooling applications, Dogonchi and Ganji^12^ explored the equations for heat transfer of a non-Newtonian fluid flow in an axisymmetric channel with a porous wall. Krishna^13^ investigated the heat transfer of alumina and copper nanofluids flowing through a stretched porous surface in a steady MHD flow. Devi and Devi^14^ investigated the MHD flow of copper-alumina/H_2_O hybrid nanofluids computationally. Krishna et al.^15^ have recently looked at radiative MHD Casson hybrid nanofluid flow across an immense exponentially improved perpendicular permeable surface. The finite element study of thermal energy inclination based on ternary hybrid nanoparticles affected by an induced magnetic field was examined by Hafeez et al.^16^. The numerical analysis of magnetic field interaction with the fully developed flow in a vertical duct was examined by Ali et al.^17^. In a vented cavity with an interior elliptic cylinder, Jamshaid et al.^18^ looked at the physical characteristics of MHD mixed convection of Ostwald-de Waele nanofluids.

Currently, a contemporary type of workings heat transfer fluids, which are called ‘’HNF’’, has grasped the response of scientists, researchers, and engineers because of its wide industrial, scientific, and technical applications such as transportation, household refrigerators, solar water heating, generator cooling, medical manufacturing, lubrication, brake fluid for cars, microfluidics, grinding and heat exchanger, etc. The HNF is a preoccupation of two different forms of NPs in the base fluid. These nanoparticles have chemical and physical properties and make it to a standardized phase by Ashorynejad and Shahriari^19^. Various numerical examinations have been passed out on the HNF as a novel concept in technology was reported by Nasrin and Alim^20^, and Ahmad et al.^21^. Shahsavar et al.^22^ investigate numerically the features of Fe3o4 -CNT/water HNF production of entropy and the heat transfer in normal convection flow inside the homocentric parallel annulus. Aman et al.^23^ showed the Na-alginate-based HNFs (Cu-Al2o3) flow in a perpendicular tube. Usman et al.^24^ reviewed the importance of time-dependent heat conductivity and nonlinear heat radiation remaining to the rotational flow of the aluminium-H2O HNF preceding an elongating sheet in the incidence of buoyancy forces and magnetic field. Analysis of activation energy and its effect on hybrid nanofluid in the presence of Hall and ion slip currents were examined by Ahmad et al.^25^. The Thermal and Solitual Transport Analysis of Blasius-Rayleigh-Stokes Flow of Hybrid Nanofluid with Convective Boundary Conditions was investigated by Qin et al.^26^. Waini et al.^27^ investigated the flow of a hybrid nanofluid toward a point of stagnation on a cylinder that was stretching and contracting. Khan et al.^28^ investigated the Radiative Mixed Convective Flow Induced by Hybrid Nanofluid across a Porous Vertical Cylinder in a Porous Media with Unusual Heat Sink/Source. Wani et al.^29^ investigated the effects of viscous dissipation and Joule heating on the flow of a micropolar hybrid nanofluid across a shrinking sheet.

There was a need to model such a fluid, which consists of micro-shaped pieces, in the last few decades. Finally, researchers' efforts resulted in the formation of a new type of fluid called a micropolar fluid. It's a group of fluids made up of microstructure molecules. Eringen^30,31^ was the first to notice this term. Origen's efforts revolutionized the study of rheologically complicated fluids by introducing the theory of the micropolar fluid, for instance, such fluids are paints, polymeric, and colloidal solutions. This term then developed into a thriving field of research. Because these fluids include micro rotation vectors as a consequence of this characteristic, they assist in the modeling of blood flow in blood vessels. Heat transmission for micropolar fluids over a permeable surface was investigated by Mirzaaghaian and Ganji^32^. They looked at how different physical characteristics affected stream function and temperature profile. Kumar et al.^33^ explored a non-Fourier heat flux model for a micropolar fluid flow through a coagulated sheet. They used shooting and R-K Fehlberg techniques to solve the modeled problem, and their results were found to be in good accord with published results. Furthermore, in this work, the buoyancy and main slip parameters were found to be rising the velocity fields functions. Ramadevi et al.^34^ examined the MHD mixed convective flow of a micropolar fluid across a stretching surface using a modified Fourier heat flux model. They observed that as the major slip parameter was increased, the velocity profile had grown while the temperature and concentration profiles declined. Kumar et al.^35^ presented simultaneous solutions for first and second-order slips of a micropolar fluid flow on a convective surface in the presence of variable heat source/sink and Lorentz force. Mehmood et al.^36^ used an internal heating phenomenon to study the micropolar Casson fluid across a stretched plate quantitatively. They looked at the effects of various physical parameters on weak and strong concentrations of temperature and velocity profiles in this study. They discovered that with comparatively low concentrations near the wall, the heat flux and skin friction decreased. Using radiation effects, Siddiqa et al.^37^ investigated a periodic spontaneous convective flow for a micropolar fluid. In this study, they used a stream function to reduce the modeled equations to a reasonable form, and then numerically solved these equations using the finite difference approach and the Keller box scheme. In comparison to earlier experiments, they achieved excellent findings. Using a magnetic field, Srinivasacharya and Bindu^38^ investigated entropy generation and heat transmission for the micropolar fluid flow inside an annulus. Using the idea of suction/injection, they were able to keep both walls moving at the same speed in this study. They solved the modeled problem using the Chebyshev spectral collection approach. In this study, the least amount of entropy creation was observed at the outer border, whereas the opposite was observed at the inner boundary, i.e., the most entropy production was observed at this surface. Kumar et al.^39^ investigated the physical features of a micropolar fluid flowing over unstable electrically conducting free convective stagnation point flow on a stretched surface. The heat source/sink and thermal radiation factors have a propensity to raise the temperature of the fluid, according to their findings. Mabood et al.^40^ evaluated the heat and mass transfer for the flow of micropolar fluid passing through a permeable channel with a stretched sheet. The fluid flow was exposed to a non-uniform source of the Soret effect, heat, and a magnetic field in this study. Ahmad et al.^41^ looked at the Cattaneo-Christov and stratification effects in the Heat Enhancement Analysis of the Hybridized Micropolar Nanofluid.

The study of heat transfer between two porous plates is currently one of the most widely researched areas. As a result, numerous kinds of research have been done to examine the heat transfer properties between two surfaces. Mustafa et al.^42^ investigated the transfer of mass and heat between two permeable surfaces. Mustafa's findings revealed a significant increase in the Nusselt number due to an increase in the Prandtl number. Alizadeh et al.^43^ explored MHD flow for a micropolar fluid through a conduit packed with NPs subjected to heat radiations. The increase in the Nusselt number values is shown to be completely reliant on the nanoparticle volume fraction and thermal radiations in this study. The hydromagnetic flow with heat transfer between two stretched surfaces was discussed by Mehmood and Ali^44^. They found that by applying a magnetic field to the system, the temperature of the system decreased. In a rotating system, Chamkha et al.^45^ studied heat transfer and nanoparticle flow between a stretched sheet and a permeable surface. They discovered that heat transmission at the surface increased the Reynolds number and the nanofluid volume fraction in both suction and injection cases. Using the thermal radiation effects, Dogonchi et al.^46^ investigated heat transfer in graphene oxide NPs over a porous channel. In this investigation, it was discovered that increasing the Reynolds number values automatically increased skin friction.

In this paper, we consider the non-Newtonian Casson tri-hybrid nano-fluid flow in the presence of the thermal radiations and Hall current effect with viscous dissipation effect which is non-compressible and laminar lapsing through an absorbent channel of breadth 2a(t). Both walls of the channel are absorbent and can move above and below with the time-dependent rate (a′(t)). Consequently, we expected that the thermal conductivity of the nanofluid increases the heat transfer efficiency. Solution of nonlinear system of ODEs will be carried out by the Bvp4c method. The important features will be corroborated with graphs for various parameters. A critical examination of these physical parameters over rotational velocity, dimensionless velocity, micro-rotations, temperature, and concentration profiles will also be presented through graphs.

## Mathematical modeling

Consider incompressible hybrid nanofluid flow through two orthogonal porous plates. The width between both plates is 2a(t) with chemical reaction and viscous dissipation effects. In comparison to the force field, the induced magnetic field is regarded to be insignificant. Here, the flow under consideration is unsteady, laminar, radiative, and viscous. As a base fluid, water is used. Between the base fluid and the nanoparticles, a thermal equilibrium exists. In Table 1 the thermophysical properties are given. Both the plates move up or down with a time-dependent rate of $a{^{\prime}}(t)$ and have the same permeability. As a consequence, as compared to a conventional heat transfer liquid, the tri-hybrid nanofluid's thermal conductivity is expected to improve heat transfer performance.

**Table 1:** Description of some dimensionless quantities.

For nanofluid^38,51^ ${\rho }_{nf}=\left(1-{\phi }_{1}\right){\rho }_{f}+{\phi }_{1}{\rho }_{s1}$ ${(\rho {C}_{p})}_{nf}=\left(1-{\phi }_{1}\right){(\rho {C}_{p})}_{f}+{\phi }_{1}{(\rho {C}_{p})}_{s1}$ ${\mu }_{nf}= \frac{{\mu }_{f}}{{\left(1-{\phi }_{1}\right)}^{2.5}}$ $\frac{{k}_{nf}}{{k}_{bf}}=\frac{{k}_{p}+(n-1){k}_{f}-(n-1){\phi }_{1}( {k}_{f}- {k}_{s1} )}{{k}_{p}+\left(n-1\right){k}_{f} + {\phi }_{1}( {k}_{f}- {k}_{s1 } )}$	For hybrid nanofluid^38^ ${\rho }_{hnf}=\left(1-{\phi }_{1}-{\phi }_{2}\right){\rho }_{f}+{\phi }_{1}{\rho }_{s1}+{\phi }_{2}{\rho }_{s2}$ ${(\rho {C}_{p})}_{hnf}=\left(1-{\phi }_{1}-{\phi }_{2}\right){(\rho {C}_{p})}_{f}+{\phi }_{1}{(\rho {C}_{p})}_{s1}+{\phi }_{2}{(\rho {C}_{p})}_{s2}$ ${\mu }_{hnf}= \frac{{\mu }_{f}}{{\left(1-{\phi }_{1}-{\phi }_{2}\right)}^{2.5}}$ $\frac{{k}_{hnf}}{{k}_{bf}}=\frac{{k}_{s2}+(n-1){k}_{bf}-(n-1){\upphi }_{2}( {k}_{bf}- {k}_{s2} )}{{k}_{s2}+\left(n-1\right){k}_{bf} + {\upphi }_{2}( {k}_{bf}- {k}_{s2 } )}$ where $\frac{{k}_{bf}}{{k}_{f}}=\frac{{k}_{s1}+(n-1){k}_{f}-(n-1){\upphi }_{1}( {k}_{f}- {k}_{s1} )}{{k}_{s1}+\left(n-1\right){k}_{f} + {\upphi }_{1}( {k}_{f}- {k}_{s1 } )}$	For tri-hybrid nanofluid^52–54^ ${\rho }_{\mathrm{trihnf}}=\left(1-{\phi }_{1}-{\phi }_{2}-{\phi }_{3}\right){\rho }_{f}+{\phi }_{1}{\rho }_{s1}+{\phi }_{2}{\rho }_{s2}+{\phi }_{3}{\rho }_{s3}$ ${(\rho {C}_{p})}_{trihnf}=\left(1-{\phi }_{1}-{\phi }_{2}-{\phi }_{3}\right){(\rho {C}_{p})}_{f}+{\phi }_{1}{(\rho {C}_{p})}_{s1}+{\phi }_{2}{(\rho {C}_{p})}_{s2}+{\phi }_{3}{(\rho {C}_{p})}_{s3}$ ${\mu }_{trihnf}= \frac{{\mu }_{f}}{{\left(1-{\phi }_{1}-{\phi }_{2}-{\phi }_{3}\right)}^{2.5}}$ $\frac{{k}_{trihnf}}{{k}_{tf}}=\frac{{k}_{s3}+(n-1){k}_{tf}-(n-1){\upphi }_{3}( {k}_{tf}- {k}_{s3} )}{{k}_{s3}+\left(n-1\right){k}_{tf} + {\upphi }_{3}( {k}_{tf}- {k}_{s3 } )}$ where $\frac{{k}_{tf}}{{k}_{bf}}=\frac{{k}_{s2}+(n-1){k}_{bf}-(n-1){\upphi }_{2}( {k}_{bf}- {k}_{s2} )}{{k}_{s2}+\left(n-1\right){k}_{bf} + {\upphi }_{2}( {k}_{bf}- {k}_{s2 } )}$ where $\frac{{k}_{bf}}{{k}_{f}}=\frac{{k}_{s1}+(n-1){k}_{f}-(n-1){\upphi }_{1}( {k}_{f}- {k}_{s1} )}{{k}_{s1}+\left(n-1\right){k}_{f} + {\upphi }_{1}( {k}_{f}- {k}_{s1 } )}$

**Figure 1:**
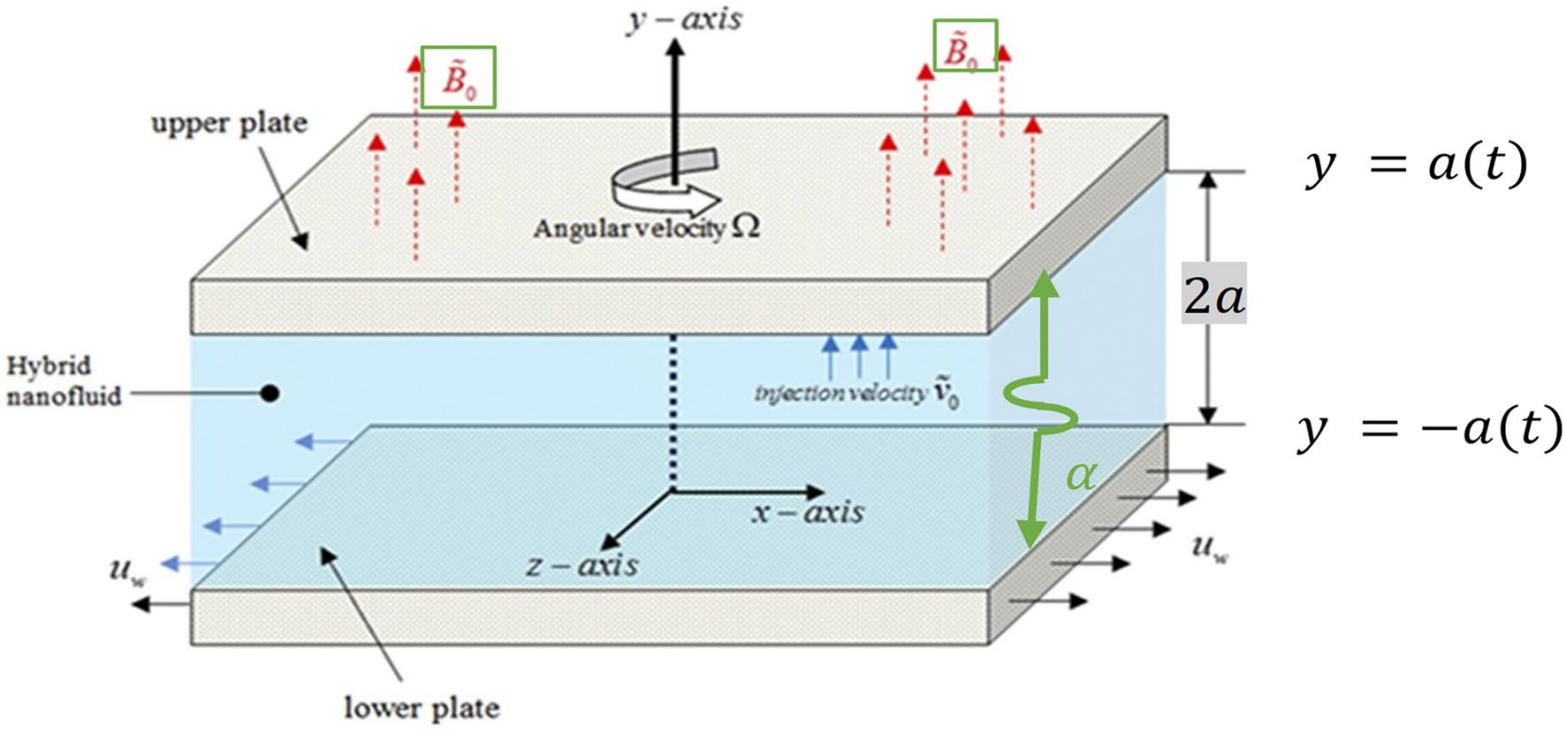
The geometry of the problem.

The geometry of the problem (shown in Fig. 1) depicts a rectangular coordinates framework with the origin at the middle of the plates and permeable walls that allow fluid to enter and exit throughout repeated expansion and contraction. Furthermore, the y-axis has been set perpendicular to the plate's walls. Under these suppositions, the Navier–Stokes equations are composed as in^11,47^, and^48^.

Continuity equations,

1 $${\widehat{u}}_{x}+{\widehat{v}}_{y}=0,$$

Momentum equations,

2 $${\widehat{u}}_{t}+\widehat{u}{\widehat{u}}_{x}+\widehat{v}{\widehat{u}}_{y}+2\Omega \widehat{w}= -\frac{1}{{\rho }_{trihnf}}{P}_{x}+\left(1+\frac{1}{\beta }\right){\upsilon }_{tryhnf}\left({\widehat{u}}_{xx}+{\widehat{u}}_{yy}\right)+\frac{k}{{\rho }_{trihnf}}{N}_{y}-\frac{1}{{\rho }_{trihnf}}\frac{{\sigma }_{f}{\left({\beta }_{0}\left(t\right)\right)}^{2}}{1+{m}^{2}}\left(\widehat{u}+m\widehat{w}\right),$$

3 $${\widehat{v}}_{t}+\widehat{u}{\widehat{v}}_{x}+\widehat{v}{\widehat{v}}_{y}= -\frac{1}{{\rho }_{trihnf}}{P}_{y}+\left(1+\frac{1}{\beta }\right){\upsilon }_{trihnf}\left({\widehat{v}}_{xx}+{\widehat{v}}_{yy}\right),$$

4 $${\widehat{w}}_{t}+\widehat{u}{\widehat{w}}_{x}+\widehat{v}{\widehat{w}}_{y}-2\Omega \widehat{u}= \left(1+\frac{1}{\beta }\right){\upsilon }_{trihnf}\left({\widehat{w}}_{xx}+{\widehat{w}}_{yy}\right)+\frac{k}{{\rho }_{trihnf}}{N}_{y}-\frac{1}{{\rho }_{trihnf}}\frac{{\sigma }_{f}{\left({\beta }_{0}\left(t\right)\right)}^{2}}{1+{m}^{2}}\left(m\widehat{u}-\widehat{w}\right),$$

Micropolar flow equation,

5 $${\rho }_{trihnf}\widehat{j}\left({N}_{t}+\widehat{u}{N}_{x}+\widehat{v}{N}_{y}\right)=-k\left(2N+{\widehat{u}}_{y}-{\widehat{v}}_{x}\right)+\gamma \left({N}_{xx}+{N}_{yy}\right),$$

Temperature equation,

6 $${\widehat{T}}_{t}+\widehat{u}{\widehat{T}}_{x}+\widehat{v}{\widehat{T}}_{y}+\widehat{w}{\widehat{T}}_{z}=\frac{{k}_{trihnf}}{{\left(\rho {C}_{p}\right)}_{trihnf}}\left({\widehat{T}}_{xx}+{\widehat{T}}_{yy}+{\widehat{T}}_{zz}\right)-\frac{1}{{\left(\rho {C}_{p}\right)}_{trihnf}}{q}_{ry},$$

where the angular velocity is denoted by $\Omega$, the magnetic field strength is denoted by ${\beta }_{0}$, P is pressure, $\widehat{j}$ is the micro inertial per unit mass, $\gamma$ is the spin gradient viscosity, and the temperature is denoted by $\widehat{T}$, and the radiative heat flux is denoted by ${q}_{r}$. N represents the micro rotation angular velocity. We use the Resseland approximation to simplify ${q}_{r}$ as in^49^

7 $${q}_{r}=\left(\frac{4{\sigma }^{*}}{3{k}^{*}}\right),$$

In the above equation, ${\sigma }^{*}$ represents the Stefan Boltzmann constant such that ${\sigma }^{*}=5.66970\times {10}^{-8}\;\;\text{W{m}}^{-2}{\text{K}}^{-4}$, whereas ${k}^{*}$ represents the mean absorption coefficient. Accordingly, Eq. (7) is reduced to the following form:

8 $${\widehat{T}}_{t}+\widehat{u}{\widehat{T}}_{x}+\widehat{v}{\widehat{T}}_{y}+\widehat{w}{\widehat{T}}_{z}=\frac{{k}_{trihnf}}{{\left(\rho {C}_{p}\right)}_{trihnf}}\left({\widehat{T}}_{xx}+{\widehat{T}}_{yy}+{\widehat{T}}_{zz}\right)-\frac{16{\sigma }^{*}{{(T}_{2})}^{3}}{3{k}^{*}{\left(\rho {C}_{p}\right)}_{trihnf}}{\widehat{T}}_{yy},$$

where ${\rho }_{trihnf}$, ${\upsilon }_{trihnf}$, ${\left(\rho \right)}_{trihnf}$, and ${k}_{trihnf}$, represent the density, kinematic viscosity, heat capacitance, and thermal conductivity of tri-hybrid nanofluid, respectively. The preparation or method employed for preparing tri hybrid nanofluid can be seen in Ramadhan et al.^50^. For micropolar NPs, these terms are defined in Table 1.

In the above table ${\rho }_{nf}$, ${\mu }_{nf}$, and ${k}_{nf}$ are the density, viscosity, and thermal conductivity of nanofluid, respectively, ${\phi }_{1}$, ${\phi }_{2}$*,* and ${\phi }_{3}$ are the volume fraction of first, second, and third nanoparticles, ${\rho }_{f}$ is the density of the base fluid, ${\rho }_{s1}$, ${\rho }_{s2}$ and ${\rho }_{s3}$ are the density of first, second, and third nanoparticles, ${k}_{s1}$, ${k}_{s2}$ and ${k}_{s3}$ are the thermal conductivity of first, second, and third nanoparticles, ${\rho }_{hnf}$, and ${k}_{hnf}$ are the density, viscosity, and thermal conductivity of hybrid nanofluid and, ${\rho }_{trihnf}$, ${\mu }_{trihnf}$ and ${k}_{trihnf}$ are the density, viscosity, and thermal conductivity of tri-hybrid nanofluid, respectively.

The boundary conditions for the problem are as follows^55–57^

9 $$\begin{aligned} \mathrm{At }y &=-a\left(t\right); \widehat{u}=0, \widehat{v}= -A{a}^{{\prime}}\left(t\right), \widehat{w}= -\frac{xA{a}^{{\prime}}\left(t\right)}{a\left(t\right)}, \widehat{T}={T}_{1}, N=-k{\widehat{u}}_{y}, \\ \mathrm{At }y&=a\left(t\right); \widehat{u}=0, \widehat{v}= A{a}^{{\prime}}\left(t\right), \widehat{w}= \frac{xA{a}^{{\prime}}\left(t\right)}{a\left(t\right)}, \widehat{T}={T}_{2}, N=k{\widehat{u}}_{y},\end{aligned}$$

Here A represents the wall permeability, and the prime indicates the derivative with respect to the time t. Here ${T}_{1}$ and ${T}_{2}$ represent temperatures of the lower and upper walls of the permeable plates, respectively, with ${T}_{1}>{T}_{2}.$

We have used the following similarity transformation as in^47,58^

10 $$\xi =\frac{y}{a\left(t\right)}, \widehat{u}= -\frac{x{\upsilon }_{f}}{{a}^{2}\left(t\right)}{F}_{\xi }\left(\xi ,t\right) , \widehat{v}=\frac{{\upsilon }_{f}}{a\left(t\right)}F\left(\xi ,t\right) , \widehat{w}= -\frac{x{\upsilon }_{f}}{{a}^{2}\left(t\right)}G\left(\xi ,t\right), \theta =\frac{\widehat{T}-{T}_{2}}{{T}_{1}-{T}_{2}}, N=\frac{x{\upsilon }_{f}}{a\left(t\right)}H\left(\xi ,t\right),$$

To reduce Eqs. (2) to (8), then we introduce the following system of nonlinear ODEs:

11 $$\left(1+\frac{1}{\beta }\right){e}_{1}{F}_{\xi \xi \xi \xi }+\alpha \left(3{F}_{\xi \xi }+\xi {F}_{\xi \xi \xi }\right)+\left({F}_{\xi }{F}_{\xi \xi }-F{F}_{\xi \xi \xi }\right)+2{R}_{0}{G}_{\xi }-{\upsilon }_{f}{e}_{2}{N}_{1}{\alpha }_{1}{H}_{\xi \xi }+\frac{{e}_{2}Ha}{1+{m}^{2}}\left(m{G}_{\xi }-{F}_{\xi \xi }\right)-{\alpha }_{1}{F}_{\xi \xi t} =0,$$

12 $$\left(1+\frac{1}{\beta }\right){e}_{1}{G}_{\xi \xi }+\alpha \left(G-\xi {G}_{\xi }\right)+\left({F}_{\xi }G-F{G}_{\xi }\right)-2{R}_{0}{F}_{\xi }+{\upsilon }_{f}{e}_{2}{N}_{1}{\alpha }_{1}{H}_{\xi }-\frac{{e}_{2}Ha}{1+{m}^{2}}\left(G-m{F}_{\xi }\right)-{\alpha }_{1}{G}_{t} =0,$$

Micropolar flow equation

13 $${e}_{2}\left({e}_{3}+\frac{{N}_{1}}{2}\right){H}_{\xi \xi }+\alpha \left(H-\xi {H}_{\xi }\right)+\left({F}_{\xi }H-F{H}_{\xi }\right)-2{\upsilon }_{f}{e}_{2}{N}_{1}\frac{{\alpha }_{1}}{\widehat{j}}H++{e}_{2}{N}_{1}\frac{1}{\widehat{j}}{F}_{\xi \xi }-{\alpha }_{1}{H}_{t}=0,$$

14 $$\left(1+\frac{4}{3}{e}_{5}{R}_{d}\right){\theta }_{\xi \xi }+{P}_{r}{e}_{4}{e}_{5}\left(\alpha \xi -F\right){\theta }_{\xi }-{\alpha }_{1}{\theta }_{t}=0,$$

where $\alpha =\frac{a\dot{a}}{{\upsilon }_{f}}$ is the expansion /contraction parameter, at this stage, we shall also declare that when $\alpha >0$, there will be expansion flow at the upper surface, and whenever $\alpha <0$, there will be contraction flow at the same surface. Also, note that^48^,

$${e}_{1}=\frac{{\upsilon }_{trihnf}}{{v}_{f}}, {e}_{2}=\frac{{\rho }_{f}}{{\rho }_{trihnf}}, {e}_{3}=\frac{{\mu }_{trihnf}}{{\mu }_{f}}, {e}_{4}=\frac{{\left(\rho {C}_{p}\right)}_{\mathrm{trihnf}}}{({\rho {C}_{p})}_{f}}, {e}_{5}=\frac{{k}_{f}}{{k}_{trihnf}}, {R}_{0}=\frac{\Omega {a}^{2}}{{\upsilon }_{f}},$$

$$Ha=\frac{{\sigma }_{f}{\beta }_{0}^{2}{a}^{2}}{{\mu }_{f}}, {N}_{1}=\frac{k}{{\mu }_{f}}, {P}_{r}=\frac{{\upsilon }_{f}({\rho {C}_{p})}_{f}}{{k}_{f}}, {R}_{d}= \frac{4{\sigma }^{*}{{(T}_{2})}^{3}}{{k}^{*}{k}_{f}}, {\alpha }_{1}=\frac{{a}^{2}}{{\upsilon }_{f}},$$

Here, $Ha$ denotes the magnetic parameter, ${R}_{0}$ denotes the rotation, ${N}_{1}$ denotes the coupling, ${R}_{d}$ is representing the radiation parameter, and ${P}_{r}$ is representing the Prandtl number. The thermophysical characteristics of the tri-hybrid nanofluid are expressed in Table 2.

**Table 2:** Thermophysical properties of the base fluid and the nanoparticles^58^.

Physical properties	Base fluid		Nanoparticles	
	Water	Fe_3_O_4_	Al_2_O_3_	TiO_2_
${C}_{p}$(j/Kg K)	2415.0	670	765	686.2
$\rho$(kg/m^3^)	1114.0	5180	3970	4250
K (W/mK)	0.252	9.7	40	8.9538

Whereas the boundary conditions are

$$\mathrm{At }\xi =-1, {F}_{\xi }=0, F=-{R}_{e}, G=-{R}_{e}, \theta =1, H\left(-1\right)=0,$$

15 $$\xi =1, {F}_{\xi }=0, F={R}_{e}, G={R}_{e}, \theta =0, H\left(1\right)=0,$$

where ${R}_{e}=\frac{A{a}^{{\prime}}a}{{\upsilon }_{f}}$ represents the Reynolds number.

By following Majdalani et al.^59^, and Uchida & Aoki^56^ we have considered the case when α is a constant, and in this case, ${\theta }_{t}={F}_{\xi \xi t}={G}_{t}=0$ so that F = F($\xi$) and θ = θ($\xi$).

Thus, we have the following equations,

16 $$\left(1+\frac{1}{\beta }\right){e}_{1}{F}_{\xi \xi \xi \xi }+\alpha \left(3{F}_{\xi \xi }+\xi {F}_{\xi \xi \xi }\right)+\left({F}_{\xi }{F}_{\xi \xi }-F{F}_{\xi \xi \xi }\right)+2{R}_{0}{G}_{\xi }-{\upsilon }_{f}{e}_{2}{N}_{1}{\alpha }_{1}{H}_{\xi \xi }+\frac{{e}_{2}Ha}{1+{m}^{2}}\left(m{G}_{\xi }-{F}_{\xi \xi }\right) =0,$$

17 $$\left(1+\frac{1}{\beta }\right){e}_{1}{G}_{\xi \xi }+\alpha \left(G-\xi {G}_{\xi }\right)+\left({F}_{\xi }G-F{G}_{\xi }\right)-2{R}_{0}{F}_{\xi }+{\upsilon }_{f}{e}_{2}{N}_{1}{\alpha }_{1}{H}_{\xi }-\frac{{e}_{2}Ha}{1+{m}^{2}}\left(G-m{F}_{\xi }\right) =0,$$

Micropolar flow equation,

18 $${e}_{2}\left({e}_{3}+\frac{{N}_{1}}{2}\right){H}_{\xi \xi }+\alpha \left(H-\xi {H}_{\xi }\right)+\left({F}_{\xi }H-F{H}_{\xi }\right)-2{\upsilon }_{f}{e}_{2}{N}_{1}\frac{{\alpha }_{1}}{\widehat{j}}H++{e}_{2}{N}_{1}\frac{1}{\widehat{j}}{F}_{\xi \xi }=0,$$

19 $$\left(1+\frac{4}{3}{e}_{5}{R}_{d}\right){\theta }_{\xi \xi }+{P}_{r}{e}_{4}{e}_{5}\left(\alpha \xi -F\right){\theta }_{\xi }=0,$$

Finally adjusting $f=\frac{F}{{R}_{e}}, g=\frac{G}{{R}_{e}}$ we have,

20 $$\left(1+\frac{1}{\beta }\right){e}_{1}{f}_{\xi \xi \xi \xi }+\alpha \left(3{f}_{\xi \xi }+\xi {f}_{\xi \xi \xi }\right)+{R}_{e}\left({f}_{\xi }{f}_{\xi \xi }-f{f}_{\xi \xi \xi }\right)+2{R}_{0}{g}_{\xi }-{\upsilon }_{f}{e}_{2}{N}_{1}\frac{{\alpha }_{1}}{{R}_{e}}{H}_{\xi \xi }+\frac{{e}_{2}Ha}{1+{m}^{2}}\left(m{g}_{\xi }-{f}_{\xi \xi }\right) =0,$$

21 $$\left(1+\frac{1}{\beta }\right){e}_{1}{g}_{\xi \xi }+\alpha \left(g-\xi {g}_{\xi }\right)+{R}_{e}\left({f}_{\xi }g-f{g}_{\xi }\right)-2{R}_{0}{f}_{\xi }+{\upsilon }_{f}{e}_{2}{N}_{1}\frac{{\alpha }_{1}}{{R}_{e}}{H}_{\xi }-\frac{{e}_{2}Ha}{1+{m}^{2}}\left(g-m{f}_{\xi }\right) =0,$$

Micropolar flow equation,

22 $${e}_{2}\left({e}_{3}+\frac{{N}_{1}}{2}\right){H}_{\xi \xi }+\alpha \left(H-\xi {H}_{\xi }\right)+{R}_{e}\left({f}_{\xi }H-f{H}_{\xi }\right)-2{\upsilon }_{f}{e}_{2}{N}_{1}\frac{{\alpha }_{1}}{\widehat{j}}H+{e}_{2}{N}_{1}\frac{{R}_{e}}{\widehat{j}}{f}_{\xi \xi }=0,$$

Temperature equation,

23 $$\left(1+\frac{4}{3}{e}_{5}{R}_{d}\right){\theta }_{\xi \xi }+{P}_{r}{e}_{4}{e}_{5}\left(\alpha \xi -{R}_{e}f\right){\theta }_{\xi }=0,$$

With boundary conditions

$$\mathrm{At }\xi =-1, {f}_{\xi }=0, f=-1, g=-1, \theta =1, H\left(-1\right)=0,$$

24 $$\xi =1, {f}_{\xi }=0, f=1, g=1, \theta =0, H\left(1\right)=0,$$

### Quantities of engineering interest

Nusselt number and SFC at both porous walls are computed coefficients that are of engineering interest and are computed in this section.

#### Skin friction coefficients (SFC)

The SFC of the upper and lower plates represents as ${C}_{f1}$ and ${C}_{f-1}$ and expressed as

25 $${C}_{f-1}=\frac{{\varsigma }_{z} \left |_{\xi =-1}\right.}{{\rho }_{f}{\left(a{^{\prime}}{A}_{1}\right)}^{2}}, \;\; and \;\; {C}_{f1}=\frac{{\varsigma }_{z} \left |_{\xi =-1}\right.}{{\rho }_{f}{\left(a{^{\prime}}{A}_{1}\right)}^{2}},$$

where ${\varsigma }_{z}$ denotes the shear stress at lower and upper plates in the radial direction, respectively,

${\varsigma }_{z} \left |_{\xi =1}= -\left({\mu }_{trihnf}+\frac{{P}_{y}}{\sqrt{2\pi }}\right)\left(\frac{\partial \widehat{u}}{\partial y}\right)\right |_{\xi =-1}$, and ${\varsigma }_{z}\left |_{\xi =1}= \left({\mu }_{trihnf}+\frac{{P}_{y}}{\sqrt{2\pi }}\right)\left(\frac{\partial \widehat{u}}{\partial y}\right)\right |_{\xi =1}$. This implies after using similarity transformation we get

26 $${C}_{f-1}= \frac{\left({\left(1-{\phi }_{1}-{\phi }_{2}-{\phi }_{3}\right)}^{-2.5}\right)}{{R}_{r}}\left(1+\frac{1}{\beta {e}_{3}{\mu }_{f}}\right){f}^{{{\prime}}{^{\prime}}}\left(-1\right), \;\; and \;\; {C}_{f1}= \frac{\left({\left(1-{\phi }_{1}-{\phi }_{2}-{\phi }_{3}\right)}^{-2.5}\right)}{{R}_{r}}\left(1+\frac{1}{\beta {e}_{3}{\mu }_{f}}\right){f}^{{^{\prime}}{^{\prime}}}\left(1\right),$$

where ${R}_{r}=\frac{a}{x}{R}_{e}$ denotes the Reynold number.

#### Nusselt number

The heat transfer rate (Nusselt number) calculations at the bottom and upper discs are given as ${N}_{u} |_{\xi=-1}$ and ${N}_{u} |_{\xi=1}$, respectively.

27 $${N}_{u}\left |_{\xi =-1}=\frac{2a{s}_{z}}{{k}_{f}\left({T}_{1}-{T}_{2}\right)}\right |_{\xi =-1}, \;\; \mathrm{ and }\;\;{N}_{u}\left |_{\xi =1}=\frac{2a{s}_{z}}{{k}_{f}\left({T}_{1}-{T}_{2}\right)}\right |_{\xi =1}.$$

Here ${s}_{z}$ is the heat flux, which is followed as,

${s}_{z} \left |_{\xi =-1}= -\left({k}_{trihnf}+\frac{16{\sigma }^{*}{{(T}_{2})}^{3}}{{3k}^{*}}\right)\left(\frac{\partial T}{\partial z}\right) \right | _{\xi =-1}, and {s}_{z}\left |_{\xi =1}= -\left({k}_{trihnf}+\frac{16{\sigma }^{*}{{(T}_{2})}^{3}}{{3k}^{*}}\right)\left(\frac{\partial T}{\partial z}\right) \right |_{\xi =1}$. This implies after using similarity transformation we get,

28 $${N}_{u}\left |_{\xi =-1}=\frac{1}{{e}_{5}}\left(1+\frac{4}{3}{e}_{5}{R}_{d}\right) {\theta }^{{\prime}}\left(-1\right),\;\;\mathrm{ and }\;\;{N}_{u}\right |_{\xi =1}=\frac{1}{{e}_{5}}\left(1+\frac{4}{3}{e}_{5}{R}_{d}\right) {\theta }^{{\prime}}\left(1\right).$$

## Numerical method

To solve the governing system of partial differential Eqs. (2)–(8), the auxiliary similarity transformation variables (10) are employed in Eqs. (2)–(8), the governing boundary value problem (2)–(8) is reduced into a system of ODEs (16)–(19). By adjusting $f=\frac{F}{{R}_{e}}, g=\frac{G}{{R}_{e}}$ into a system of ODEs (16)–(19), we get Eqs. (20)–(23).

**Figure 2:**
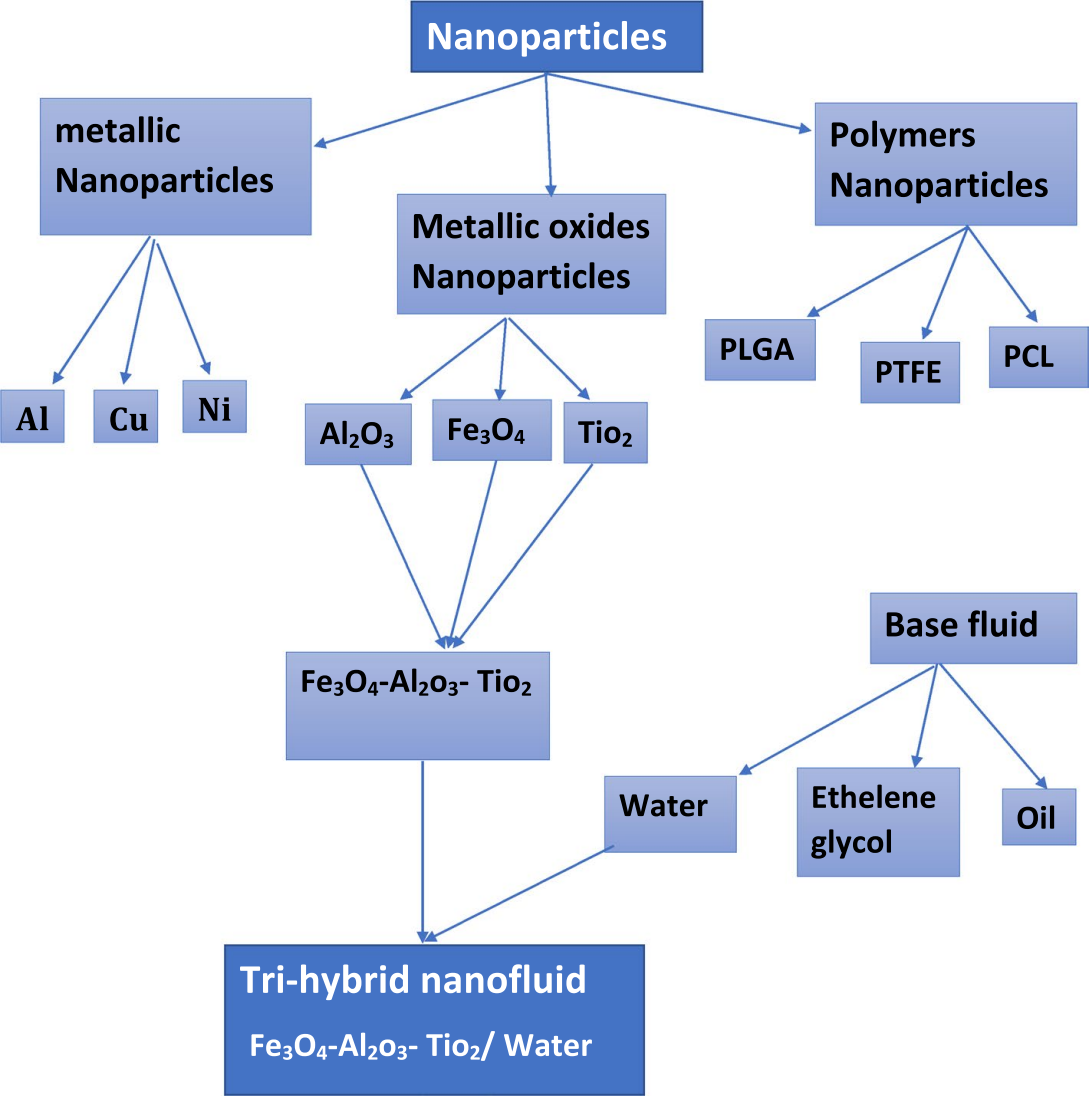
Flow chart of tri-hybrid nanofluid.

The nonlinear system of ODEs (20)–(23) arising from mathematical modeling of the physical system of nanofluid flow is difficult to solve analytically. Therefore, the bvp4c function from MATLAB is used to attain the numerical solution for titanium dioxide-water (TiO_2_-H_2_O), iron oxide—titanium dioxide/water (Fe_3_O_4_- TiO_2_/H_2_O), and iron oxide -alumina-titanium dioxide/water (Fe_3_O_4_-Al_2_O_3_-TiO_2_/H_2_O). Figure 2 represents the considered tri-hybrid nanofluid flow chart. Conclusions are observed graphically with a focus on the mathematical model’s main characteristics and their result on velocity, temperature, and microrotation velocity disseminations.

To use the bvp4c method first convert the system of ordinary differential Eqs. (20)–(23) into a system of first-order ODEs.

29 $$f=y\left(1\right), {f}^{{\prime}}=y\left(2\right), {f}^{{{\prime}}{{\prime}}}=y\left(3\right),$$

$${e}_{1}=\frac{{\upsilon }_{trihnf}}{{v}_{f}}, {e}_{2}=\frac{{\rho }_{f}}{{\rho }_{trihnf}}, {e}_{3}=\frac{{\mu }_{trihnf}}{{\mu }_{f}}, {e}_{4}=\frac{{\left(\rho {C}_{p}\right)}_{trihnf}}{({\rho {C}_{p})}_{f}}, {e}_{5}=\frac{{k}_{f}}{{k}_{trihnf}}, {R}_{0}=\frac{\Omega {a}^{2}}{{\upsilon }_{f}},$$

30 $$Ha=\frac{{\sigma }_{f}{\beta }_{0}^{2}{a}^{2}}{{\mu }_{f}}, {N}_{1}=\frac{k}{{\mu }_{f}}, {P}_{r}=\frac{{\upsilon }_{f}({\rho {C}_{p})}_{f}}{{k}_{f}}, {R}_{d}= \frac{4{\sigma }^{*}{T}_{2}}{{k}^{*}{k}_{f}}, {\alpha }_{1}=\frac{{a}^{2}}{{\upsilon }_{f}},$$

31 $${f}_{\xi \xi \xi \xi }={\left(1+\frac{1}{\beta }\right)}^{-1}\frac{1}{{e}_{1}} \left[-\alpha \left(3{f}_{\xi \xi }+\xi {f}_{\xi \xi \xi }\right)-{R}_{e}\left({f}_{\xi }{f}_{\xi \xi }-f{f}_{\xi \xi \xi }\right)-2{R}_{0}{g}_{\xi }+{\upsilon }_{f}{e}_{2}{N}_{1}\frac{{\alpha }_{1}}{{R}_{e}}{H}_{\xi \xi }-\frac{{e}_{2}Ha}{1+{m}^{2}}\left(m{g}_{\xi }-{f}_{\xi \xi }\right) \right],$$

32 $$g= y\left(5\right), {g}_{\xi }= y\left(6\right),$$

$${g}_{\xi \xi }={\left(1+\frac{1}{\beta }\right)}^{-1}\frac{1}{{e}_{1}}\left[-\alpha \left(g-\xi {g}_{\xi }\right)-{R}_{e}\left({f}_{\xi }g-f{g}_{\xi }\right)+2{R}_{0}{f}_{\xi }-{\upsilon }_{f}{e}_{2}{N}_{1}\frac{{\alpha }_{1}}{{R}_{e}}{H}_{\xi }+\frac{{e}_{2}Ha}{1+{m}^{2}}\left(g-m{f}_{\xi }\right) \right],$$

33 $$H= y\left(7\right), {H}_{\xi }= y\left(8\right),$$

34 $${H}_{\xi \xi }={\left({e}_{3}+\frac{{N}_{1}}{2}\right)}^{-1}\frac{1}{{e}_{2}}\left[-\alpha \left(H-\xi {H}_{\xi }\right)-{R}_{e}\left({f}_{\xi }H-f{H}_{\xi }\right)+2{\upsilon }_{f}{e}_{2}{N}_{1}\frac{{\alpha }_{1}}{\widehat{j}}H-{e}_{2}{N}_{1}\frac{{R}_{e}}{\widehat{j}}{f}_{\xi \xi }\right],$$

35 $$\theta = y\left(9\right), {\theta }_{\xi }= y\left(10\right),$$

36 $${\theta }_{\xi \xi }={\left(1+\frac{4}{3}{e}_{5}{R}_{d}\right)}^{-1}\left[-{P}_{r}{e}_{4}{e}_{5}\left(\alpha \xi -{R}_{e}f\right){\theta }_{\xi }\right].$$

## Result and discussion

In the present section, we elaborated the graphical results for various physical quantities under consideration. The physical variables are attained from the system of flow governing equations which are then solved in a numerical way using the bvp4c method. The graphs of the various dimensionless quantities are elaborated onto velocity and thermal fields.

**Figure 3:**
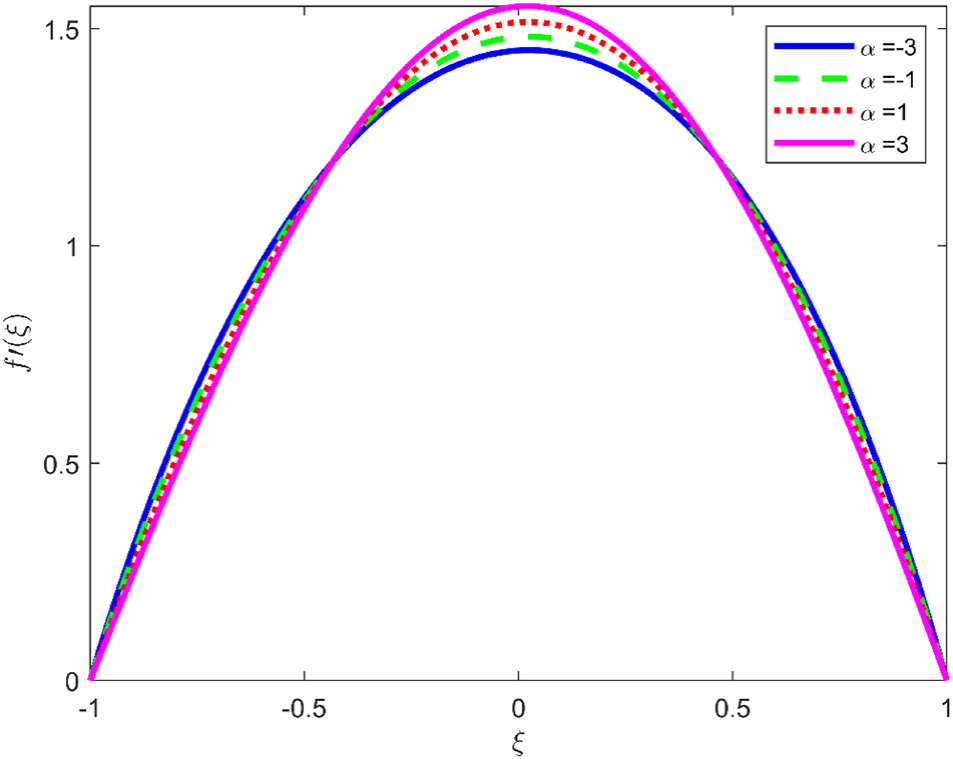
Effect of $\alpha$ upon velocity profile $f{^{\prime}}(\xi )$, for selected values of $\beta =0.2, m=1, Ha=1, {\phi }_{1}={\phi }_{2}={\phi }_{3}=0.02$, ${R}_{0}=1, S=3, {R}_{d}=1, {N}_{1}=1, {R}_{e}=-0.5, {P}_{r}=6.2.$

**Figure 4:**
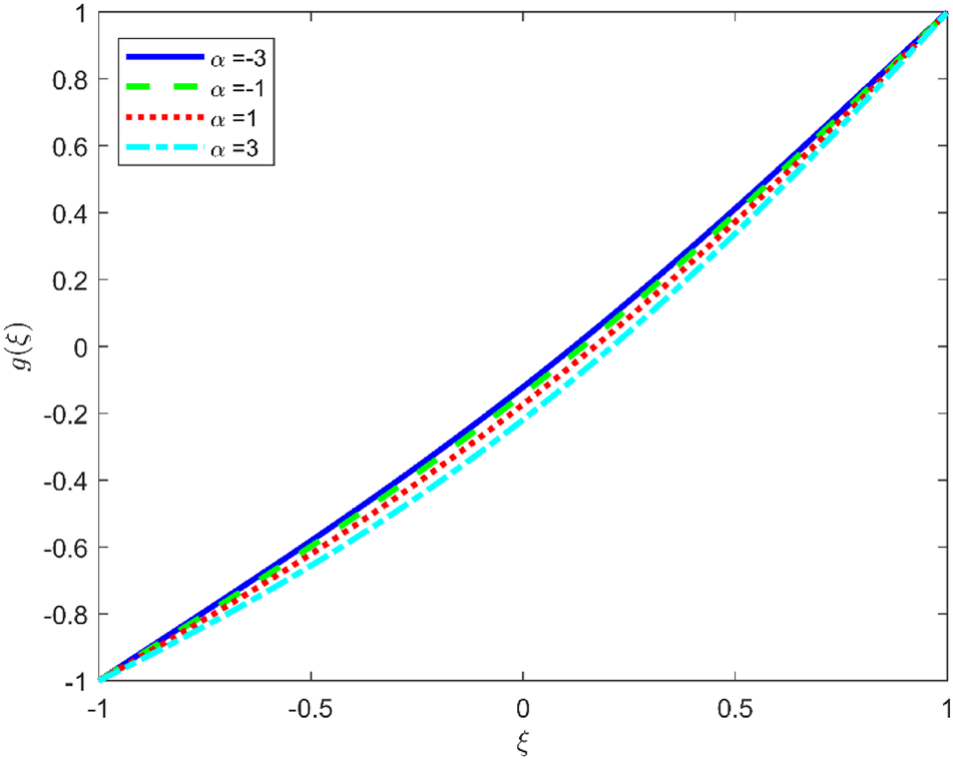
Effect of $\alpha$ upon velocity profile $g(\xi )$, for selected values of $\beta =0.2, m=1, Ha=1, {\phi }_{1}={\phi }_{2}={\phi }_{3}=0.02$, ${R}_{0}=1, S=3, {R}_{d}=1, {N}_{1}=1, {R}_{e}=-0.5, {P}_{r}=6.2.$

**Figure 5:**
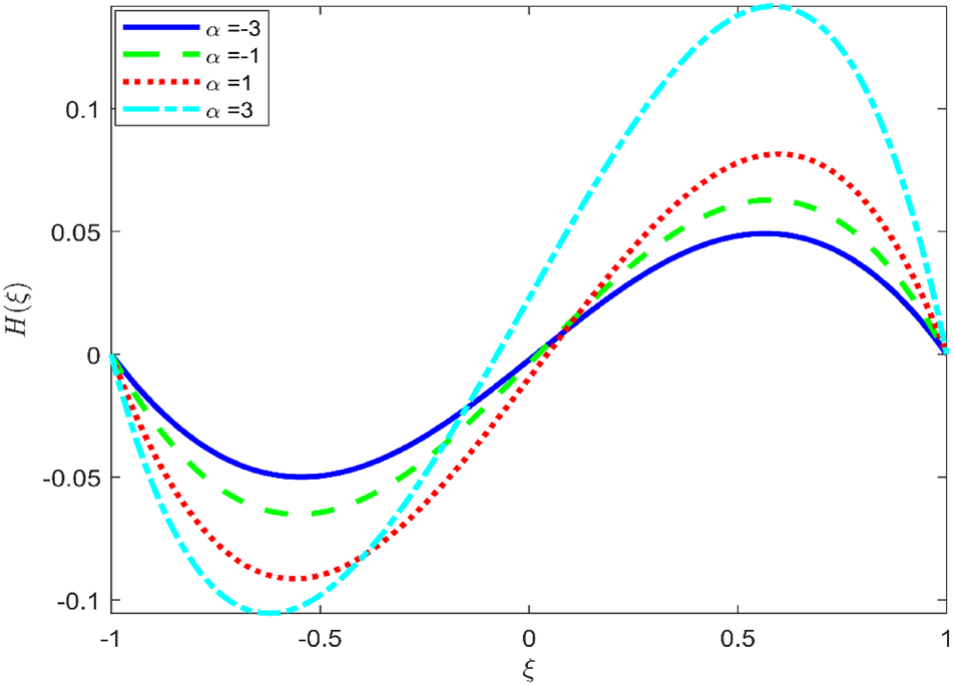
Effect of $\alpha$ upon velocity profile $H(\xi )$, for selected values of $\beta =0.2, m=1, Ha=1, {\phi }_{1}={\phi }_{2}={\phi }_{3}=0.02$, ${R}_{0}=1, S=3, {R}_{d}=1, {N}_{1}=1, {R}_{e}=-0.5, {P}_{r}=6.2.$

**Figure 6:**
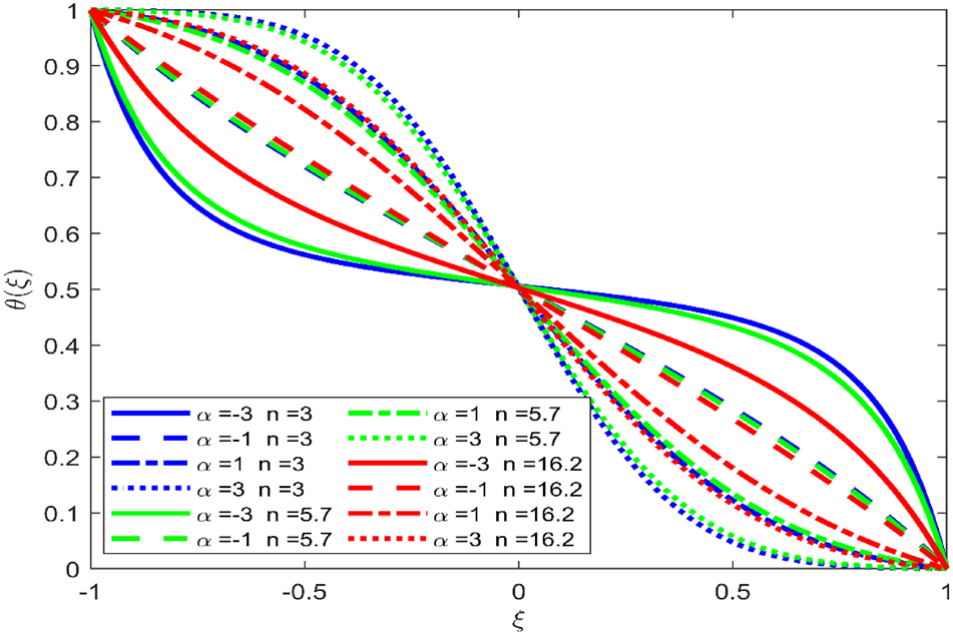
Effect of $\alpha$ upon velocity profile $\theta (\xi )$, for selected values of $\beta =0.2, m=1, Ha=1, {\phi }_{1}={\phi }_{2}={\phi }_{3}=0.02$, ${R}_{0}=1, S=3, {R}_{d}=1, {N}_{1}=1, {R}_{e}=-0.5, {P}_{r}=6.2.$

**Figure 7:**
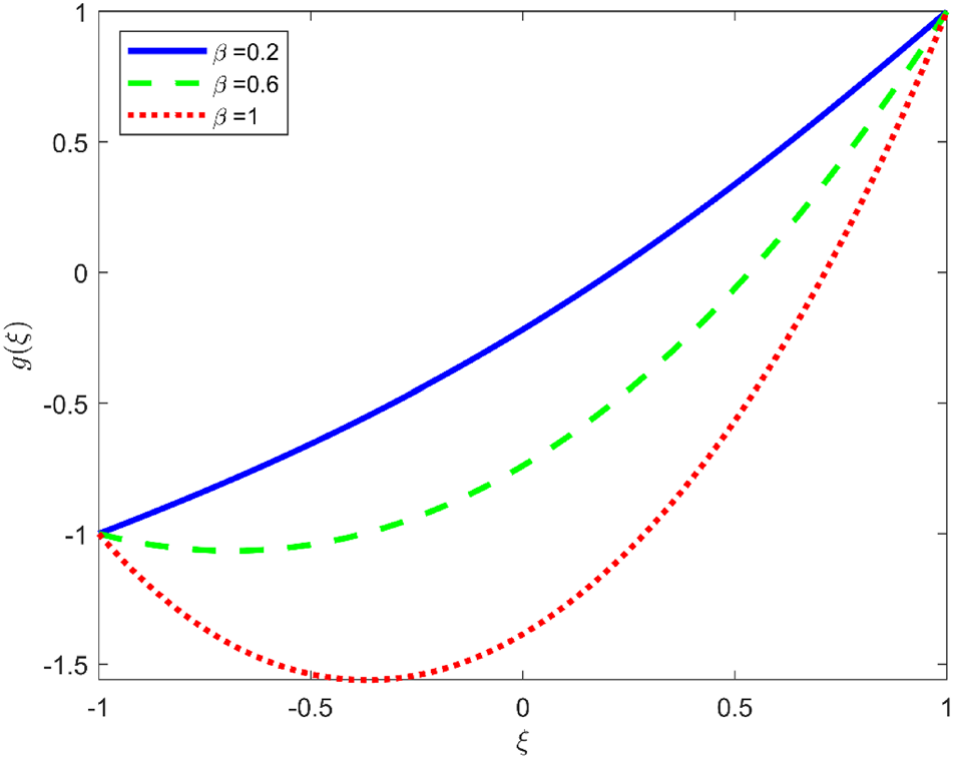
Effect of $\beta$ upon velocity profile $g(\xi )$, for selected values of $m=1, Ha=1, {\phi }_{1}={\phi }_{2}={\phi }_{3}=0.02$, ${R}_{0}=1, S=3, {R}_{d}=1, {N}_{1}=1, {R}_{e}=-0.5, {P}_{r}=6.2, \alpha =3.$

**Figure 8:**
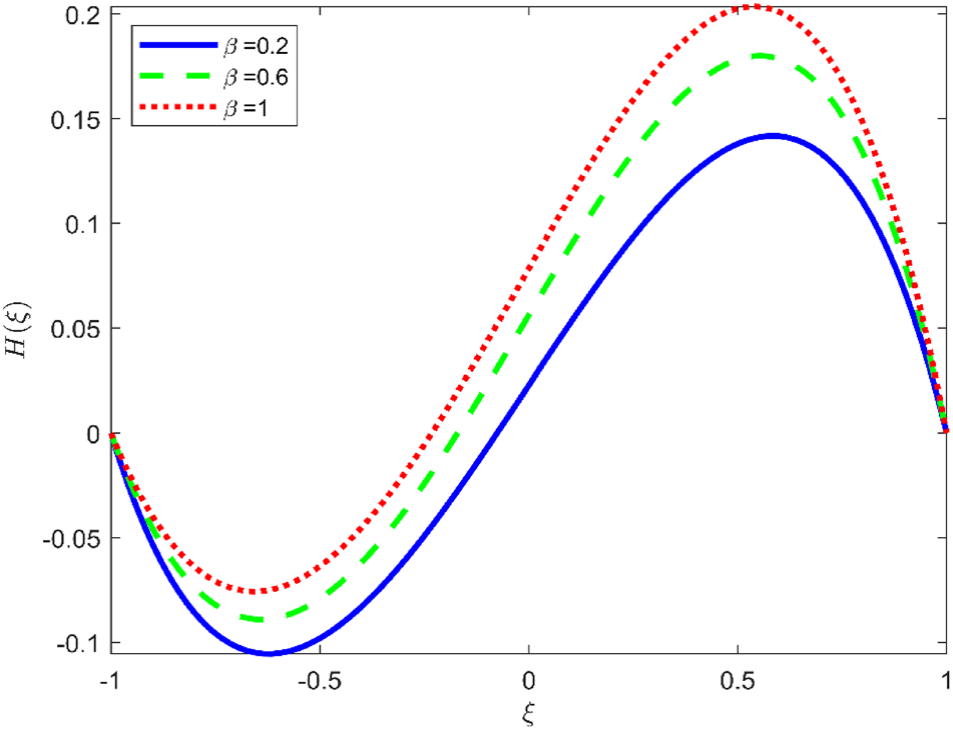
Effect of $\beta$ upon velocity profile $H(\xi )$, for selected values of $m=1, Ha=1, {\phi }_{1}={\phi }_{2}={\phi }_{3}=0.02$, ${R}_{0}=1, S=3, {R}_{d}=1, {N}_{1}=1, {R}_{e}=-0.5, {P}_{r}=6.2, \alpha =3.$

**Figure 9:**
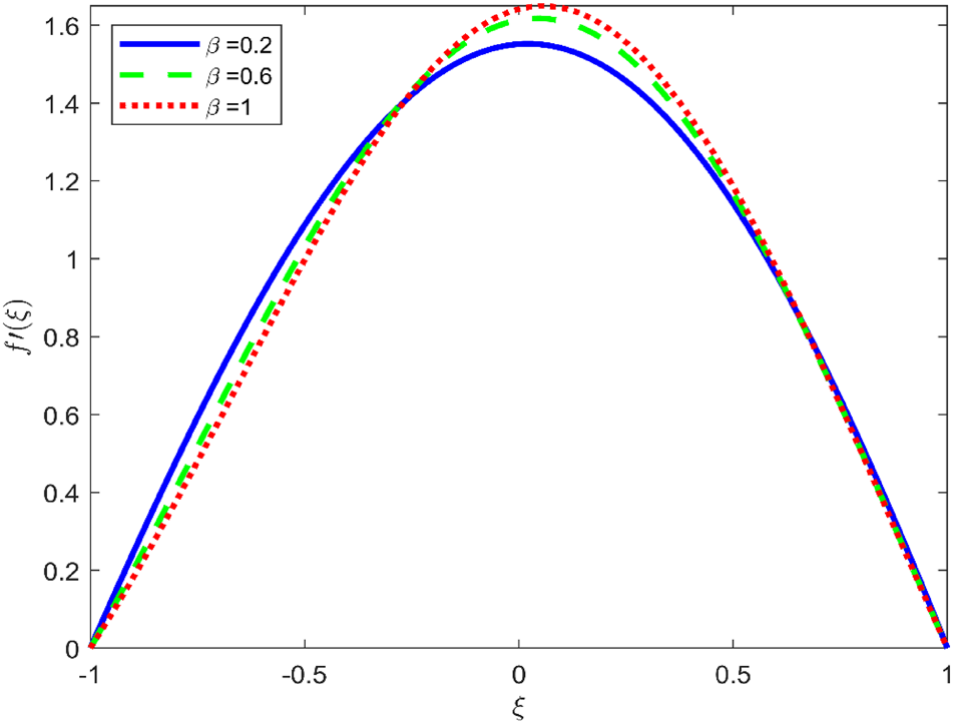
Effect of $\beta$ upon velocity profile $f{^{\prime}}(\xi )$, for selected values of $m=1, Ha=1, {\phi }_{1}={\phi }_{2}={\phi }_{3}=0.02$, ${R}_{0}=1, S=3, {R}_{d}=1, {N}_{1}=1, {R}_{e}=-0.5, {P}_{r}=6.2, \alpha =3$

**Figure 10:**
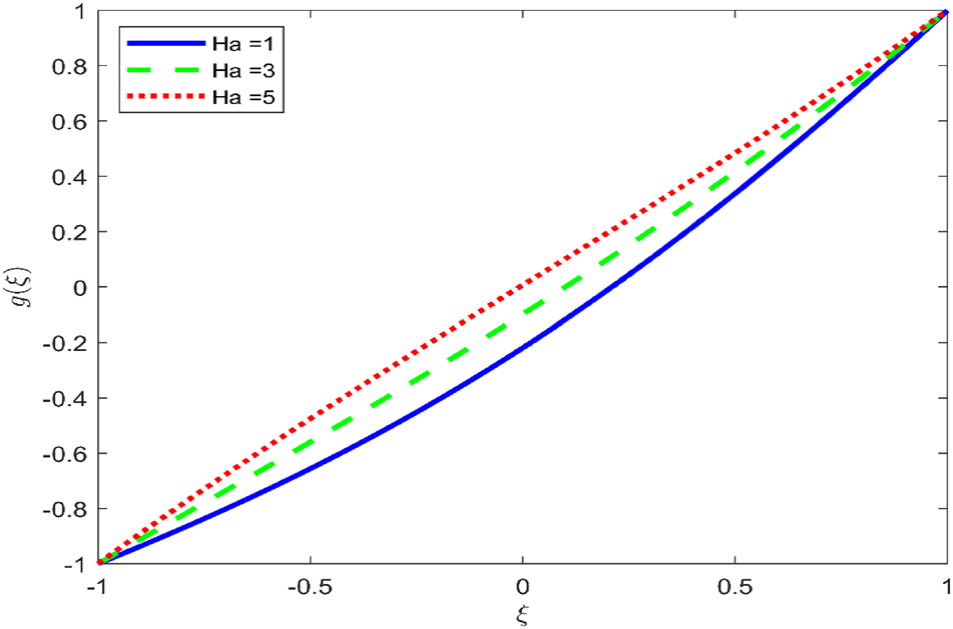
Effect of $Ha$ upon velocity profile $g(\xi )$, for selected values of $\beta =0.2, m=1, {\phi }_{1}={\phi }_{2}={\phi }_{3}=0.02$, ${R}_{0}=1, S=3, {R}_{d}=1, {N}_{1}=1, {R}_{e}=-0.5, {P}_{r}=6.2, \alpha =3$

**Figure 11:**
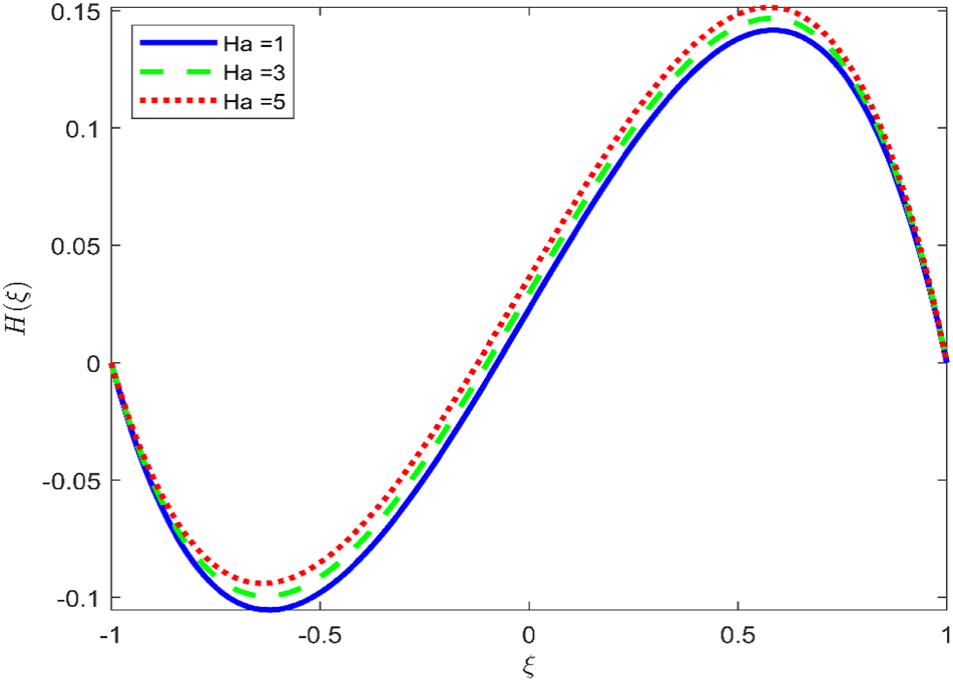
Effect of $Ha$ upon velocity profile $H(\xi )$, for selected values of $\beta =0.2, m=1, {\phi }_{1}={\phi }_{2}={\phi }_{3}=0.02$, ${R}_{0}=1, S=3, {R}_{d}=1, {N}_{1}=1, {R}_{e}=-0.5, {P}_{r}=6.2, \alpha =3.$

**Figure 12:**
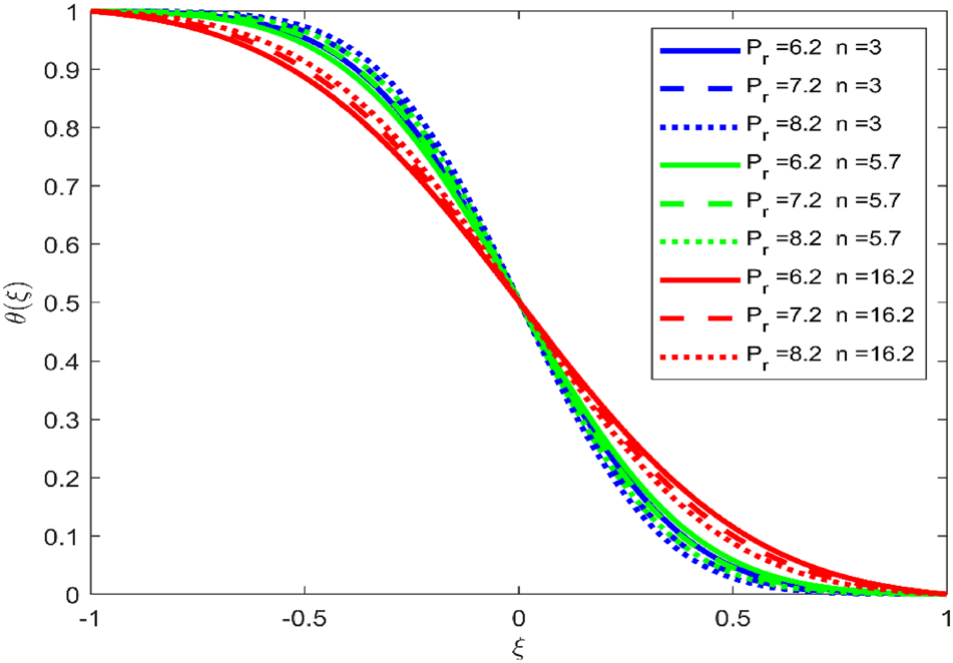
Effect of ${P}_{r}$ upon velocity profile $\theta (\xi )$, for selected values of $\beta =0.2, m=1, Ha=1, {\phi }_{1}={\phi }_{2}={\phi }_{3}=0.02$, ${R}_{0}=1, S=3, {R}_{d}=1, {N}_{1}=1, {R}_{e}=-0.5, \alpha =3.$

**Figure 13:**
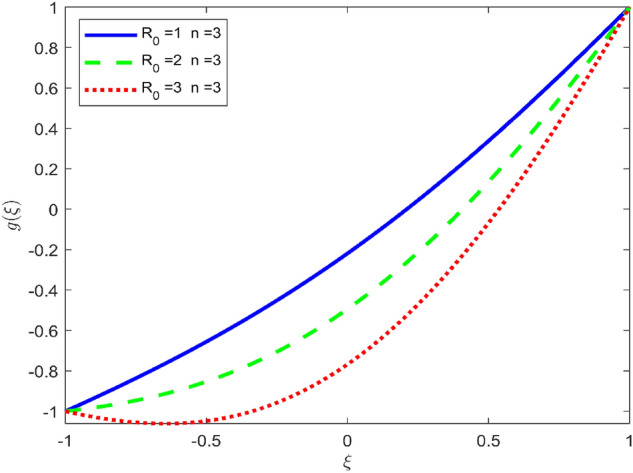
Effect of ${R}_{0}$ upon velocity profile $g(\xi )$, for selected values of $\beta =0.2, m=1, Ha=1, {\phi }_{1}={\phi }_{2}={\phi }_{3}=0.02$, $S=3, {R}_{d}=1, {N}_{1}=1, {R}_{e}=-0.5, {P}_{r}=6.2, \alpha =3$.

**Figure 14:**
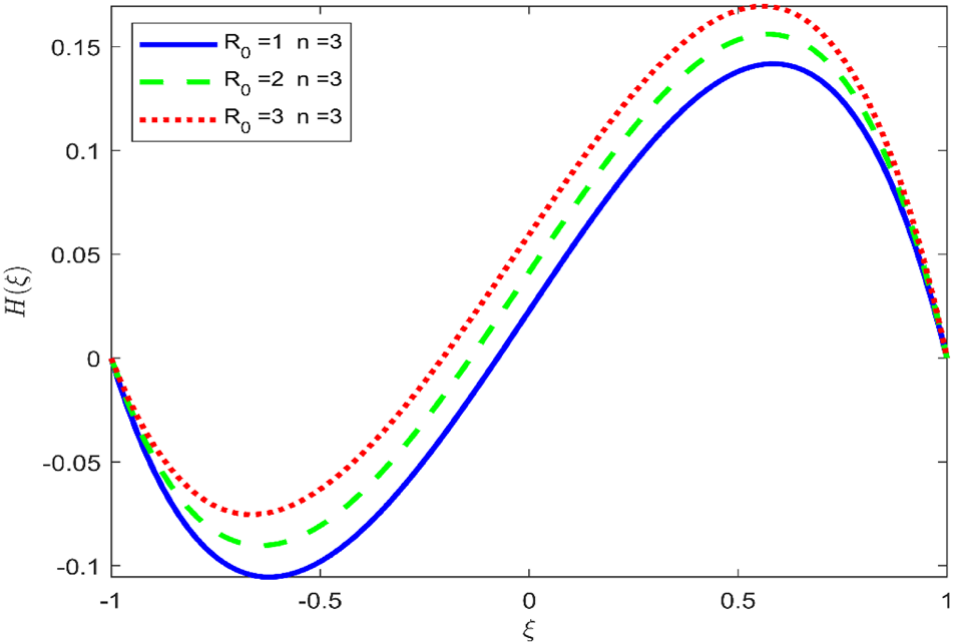
Effect of ${R}_{0}$ upon velocity profile $H(\xi )$, for selected values of $\beta =0.2, m=1, Ha=1, {\phi }_{1}={\phi }_{2}={\phi }_{3}=0.02$, ${R}_{0}=1, S=3, {R}_{d}=1, {N}_{1}=1, {R}_{e}=-0.5, {P}_{r}=6.2, \alpha =3$.

**Figure 15:**
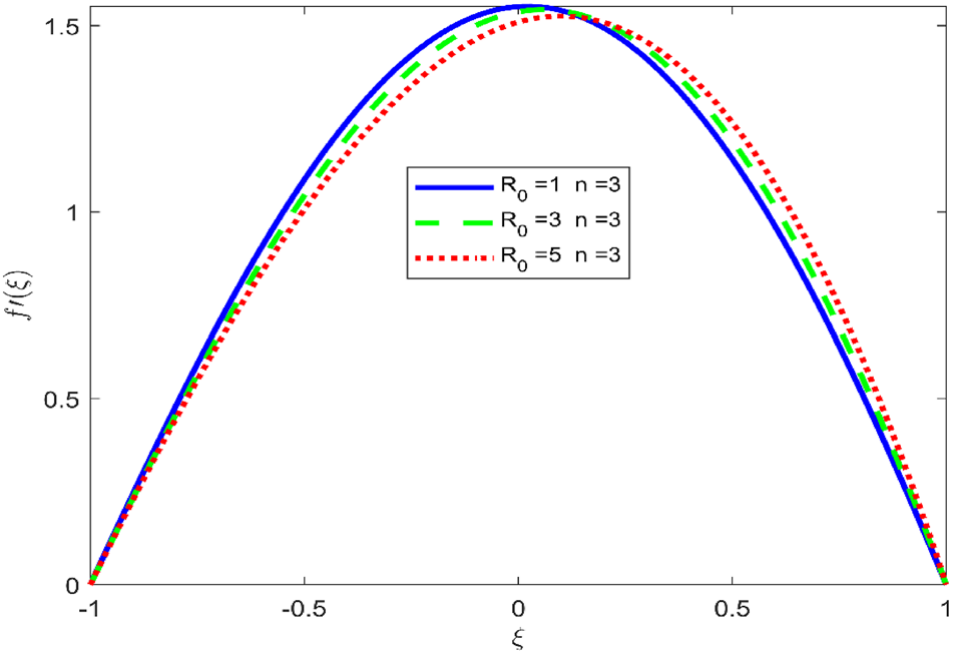
Effect of ${R}_{0}$ upon velocity profile $f{^{\prime}}(\xi )$, for selected values of $\beta =0.2, m=1, Ha=1, {\phi }_{1}={\phi }_{2}={\phi }_{3}=0.02$, $S=3, {R}_{d}=1, {N}_{1}=1, {R}_{e}=-0.5, {P}_{r}=6.2, \alpha =3$.

**Figure 16:**
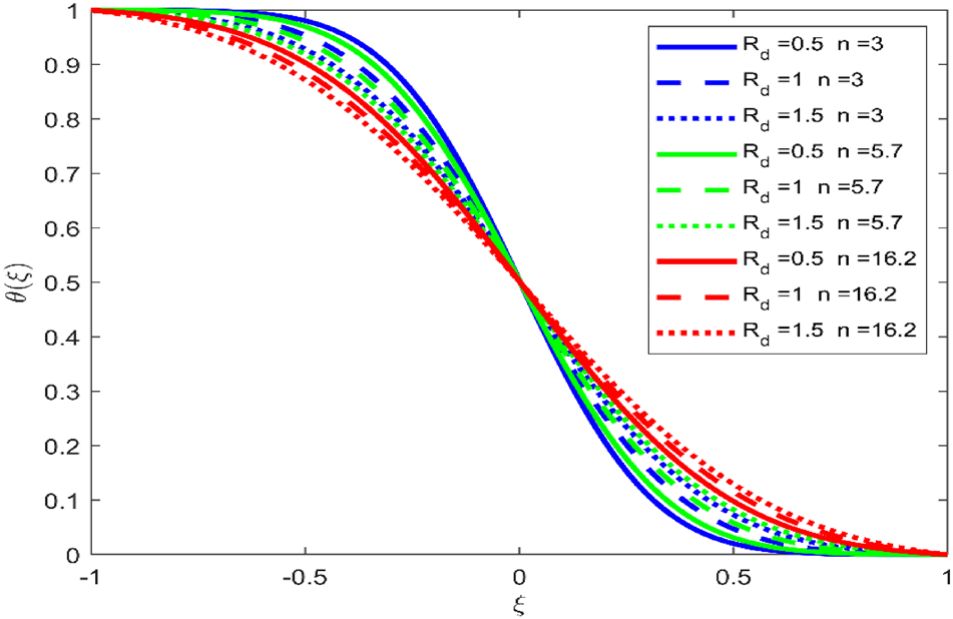
Effect of ${R}_{d}$ upon velocity profile $\theta (\xi )$, for selected values of $\beta =0.2, m=1, Ha=1, {\phi }_{1}={\phi }_{2}={\phi }_{3}=0.02$, ${R}_{0}=1, S=3, {N}_{1}=1, {R}_{e}=-0.5, {P}_{r}=6.2, \alpha =3$.

**Figure 17:**
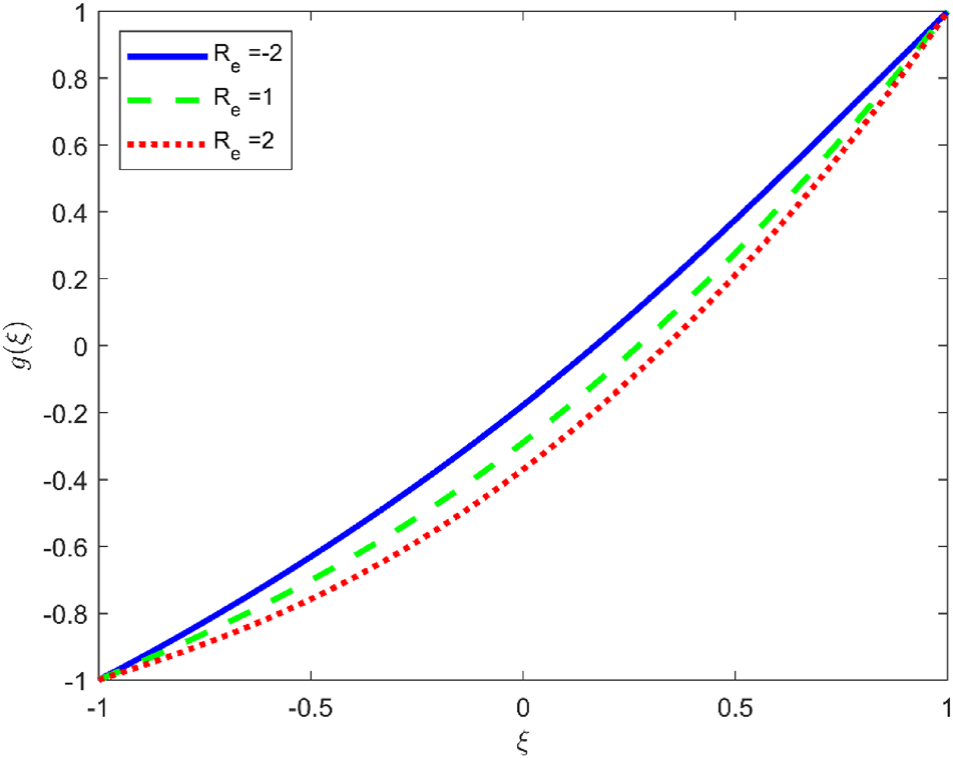
Effect of ${R}_{e}$ upon velocity profile $g(\xi )$, for selected values of $\beta =0.2, m=1, Ha=1, {\phi }_{1}={\phi }_{2}={\phi }_{3}=0.02$, ${R}_{0}=1, S=3, {R}_{d}=1, {N}_{1}=1, {P}_{r}=6.2, \alpha =3$.

**Figure 18:**
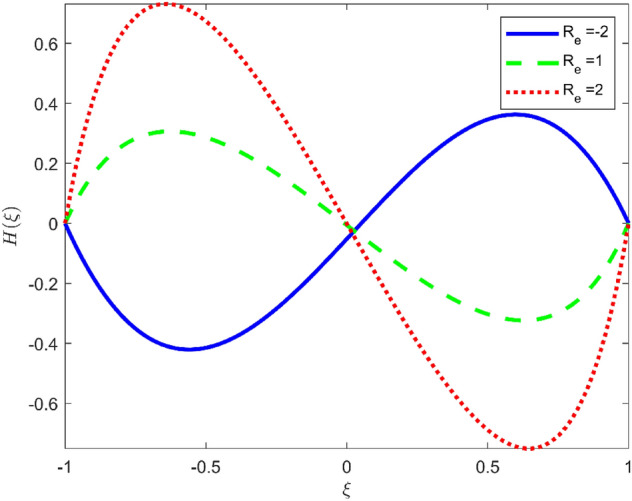
Effect of ${R}_{e}$ upon velocity profile $H(\xi )$, for selected values of $\beta =0.2, m=1, Ha=1, {\phi }_{1}={\phi }_{2}={\phi }_{3}=0.02$, ${R}_{0}=1, S=3, {R}_{d}=1, {N}_{1}=1, {P}_{r}=6.2, \alpha =3$.

**Figure 19:**
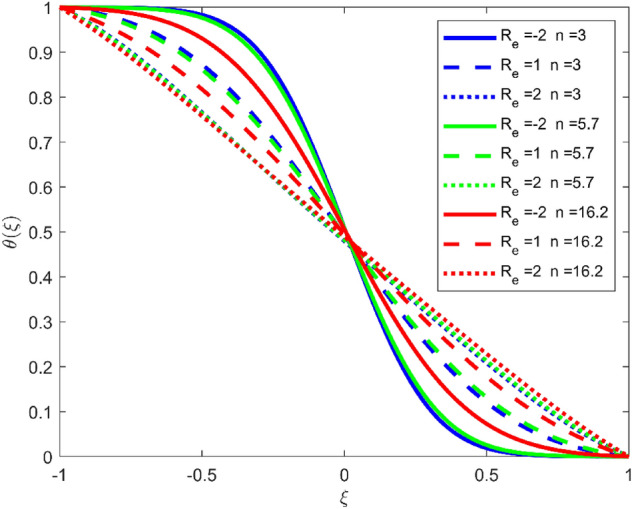
Effect of ${R}_{e}$ upon velocity profile $\theta (\xi )$, for selected values of $\beta =0.2, m=1, Ha=1, {\phi }_{1}={\phi }_{2}={\phi }_{3}=0.02$, ${R}_{0}=1, S=3, {R}_{d}=1, {N}_{1}=1, {P}_{r}=6.2, \alpha =3$.

Figures 3, 4, and 5 are established to signify the consequences of expansion/contraction parameters on velocity fields $f{^{\prime}}$, $g,$ and $H$ respectively. It is observed that as the values of $\alpha$ vary from negative to positive the velocity $f{^{\prime}}$ increases in the middle of permeable plates, rotational velocity decreases, and micropolar velocity decline at the lower plate and rises at the upper plate. Figure 6 is plotted to show the behavior of the expansion/contraction parameter on the temperature profile. It is noticed that for all three cases of shape size factor (spherical, cylindrical, laminar) when enlarging the value of $\alpha$ for expansion and contraction cases, the temperature increases at the lower plate and decreases at the upper plate. For the contraction case the increment in $S$ decreases the temperature at the upper plate and increases the temperature at the lower plate, the reverse behavior is observed for the expansion case. The graphical depiction of the $\beta$ onto the velocity flow phenomenon is sketched in Figs. 7, 8 and 9. It is observed that the increment in $\beta$ decreases the rotational velocity and increases the micropolar velocity, the effect of $\beta$ on radial velocity is of the same nature in comparison to the expansion parameter. Physically, the current figures exhibit that the fluid gets more viscous with the increasing values of $\beta$ which results in the reduction of velocities of the fluid. Moreover, it is also worth mentioning here that the case for viscous HNF can be achieved for $\beta \to \infty$, i.e., the problem will reduce to a Newtonian case. Figures 10 and 11 are plotted to show the behavior of Ha onto the velocity fields. It is observed that the increment in Ha, increases the rotational and micro rotational velocities. As the increase of the applied magnetic field produces more resistance to the flow phenomenon, which in turn declines the velocity field. Figure 12 elaborates on the thermal phenomenon against the Prandtl number. For all three cases of shapes size factor (spherical, cylindrical, laminar) the larger ${P}_{r}$ values resulted in the decrement of the temperature at the upper plate and an increase at the lower plate (see Fig. 12). This is because the Prandtl number is taken as the ratio of momentum diffusivity to thermal diffusivity and the higher values of ${P}_{r}$ decrease the thermal diffusivity which results in the reduction of temperature. For the larger value of S temperature decrease at the lower plate and increases at the upper plate. Figures 13, 14 and 15 are plotted to illustrate the pictorial representation of ${R}_{0}$ on rotational, micro rotational, and radial velocity profiles respectively. The parameter ${R}_{0}$ effects on rotational and micro rotational velocity profiles are observed to be opposite in comparison to $\beta$, and it is observed that the increment in ${R}_{0}$, increases the radial velocity at the upper plate and decreases at the lower plate. Figure 16 elaborates on the thermal phenomenon against the radiation parameter. For all three cases of shape size factor (spherical, cylindrical, laminar), it is noticed that the larger value of radiation parameter resulted in the decrement of the temperature at the lower plate and increase at the upper plate. Physically, the parameter ${R}_{d}$ enables us to increase the radiative heat transfer with its increasing values. Figures 17 and 18 are sketched to illustrate the permeable Reynolds number impact on the velocity fields. It is observed the value of ${R}_{e}$ moves from negative to positive, the rotational velocity profile decreases, and for the suction case the increment in ${R}_{e}$ decrease the micro rotational velocity at the lower plate and increase at the upper plate, the opposite behavior is observed for injection case. Figure 19 is established to signify the consequences of parameters ${R}_{e}$ and $S$ on the thermal phenomena. For all three cases of shapes size factor (spherical, cylindrical, laminar), it is noticed as the value of ${R}_{e}$ changes from negative to positive, the temperature increases at the upper plate and decreases at the lower plate. Table 3 represents variation in the effect of $\alpha$, $\beta$, Ha, and volume fractions on SFC and Nusselt numbers for suction and injection cases. For suction case ${R}_{e}<0$, it is observed that as the value of $\alpha$ moves from negative to positive the SFC and Nusselt number decreases. The increment in $\beta$ decrease in the SFC and Nusselt number is noticed. The influence of Ha and $\beta$ have opposite in nature on the SFC and Nusselt numbers. The larger value of ${\phi }_{1}$, ${\phi }_{2}$, and ${\phi }_{3}$ resulted in the decrement of SFC and increment in Nusselt number. For injection case ${R}_{e}>0$, the opposite behavior of influence of $\beta$ and Ha on the SFC and Nusselt number as compared to the suction case is observed. The increment in $\alpha$ increases the SFC and decreases the Nusselt number is noticed. The increase in volume fraction rises the SFC and Nusselt numbers. Table 4 demonstrates the influence of the ${R}_{0}$, $S$, ${R}_{d}$, and ${P}_{r}$ on the skin friction coefficient and Nusselt number for suction and injection cases. For the suction case, it is observed that the increment in ${R}_{0}$ decreases the SFC and Nusselt number. The increment in shape size factor and radiation parameter enhances the Nusselt number. The influence of Prandtl number and radiation parameter have opposite behavior on Nusselt number. By definition, thermal diffusivity is decreased by the modified Prandtl number, therefore larger ${P}_{r}$ values resulted in the decrement of the Nusselt number. For the injection case, it is noticed that the increment in ${R}_{0}$ decreases the SFC and increases the Nusselt number. The impact of the shape size factor, radiation parameter, and Prandtl number on the Nusselt number same as in the suction case. The effect of ${R}_{e}$, $\beta$, Ha, ${\upphi }_{1}$,${\upphi }_{2}$, and ${\upphi }_{3}$ on Nusselt number and SFC for contraction and expansion cases are shown in Table 5. For the contraction case, Table 5 also shows that by increasing $\beta$ and Ha, the SFC and Nusselt number rises. It is noticed that the value of ${R}_{e}$ moves from negative to positive the Nusselt number increases and for the suction/injection case the increment in ${R}_{e}$ increases the SFC. The enhancement in ${\upphi }_{1}$,${\upphi }_{2}$, and ${\upphi }_{3}$ increase the Nusselt number and decrease the SFC. For the expansion case, the impact of ${R}_{e}$, $\beta$, Ha, ${\upphi }_{1}$,${\upphi }_{2}$, and ${\upphi }_{3}$ on skin friction and Nusselt number are the same in behavior as in the contraction case. Table 6 states variation in the effect of ${R}_{0}$, $S$, ${R}_{d}$, and ${P}_{r}$ on the SFC and Nusselt number for contraction and expansion cases**.** For the contraction case, it is observed that the increment in ${R}_{0}$ decrease the SFC and Nusselt number. The increment in shape size factor and radiation parameter enhances the Nusselt number is noticed. The influence of Prandtl number and radiation parameter have opposite behavior on Nusselt number. By definition, thermal diffusivity is decreased by the modified Prandtl number, therefore larger ${P}_{r}$ values resulted in the decrement of the Nusselt number. For the expansion case, the influence of ${R}_{0}$, $S$, ${R}_{d}$ and ${P}_{r}$ on skin friction and Nusselt number are the same in the behavior as in the contraction case observed.

**Table 3:** The impact of $\alpha$, $\beta$, Ha, ${\upphi }_{1}$,${\upphi }_{2}$, and ${\upphi }_{3}$ on the SFC and Nusselt number for suction and injection case, for ${R}_{0}=1, S=3, {R}_{d}=1, {N}_{1}=1.$

$\alpha$	$\beta$	Ha	${\phi }_{1}={\phi }_{2}={\phi }_{3}$	${C}_{f-1}$ for suction case (${R}_{e}=-0.5$)	${N}_{u} \left |_{\eta =-1}\right.$ for suction case $({R}_{e}=-0.5)$	${C}_{f-1}$ for injection case $({R}_{e}=0.5)$	${N}_{u} \left | _{\eta =-1}\right.$ for injection case $({R}_{e}=0.5)$
− 2	0.2	1	2%	28.5466	5.0465	28.7159	9.9240
− 0.5				26.6857	1.3614	26.8082	4.0747
0.5				25.4620	0.4405	25.5529	1.7113
2				23.6539	0.0645	23.6974	0.3234
− 0.5	0.2			26.6857	1.3614	26.8082	4.0747
	0.4			15.716	1.3505	15.8601	4.1105
	0.7			11.0645	1.3398	11.2078	4.1470
	1			9.2097	1.3328	9.3678	4.1716
		2		26.6029	1.3587	26.7198	4.0829
		4		26.4289	1.3537	26.5329	4.0989
		6		26.2456	1.3488	26.3353	4.1141
− 0.5	0.2	1	3%	23.0584	1.4021	23.1647	4.1035
			4%	19.8505	1.4434	19.9416	4.1288
			5%	17.0228	1.4854	17.0999	4.1537
			6%	14.5389	1.5279	14.6033	4.1774

**Table 4:** The impact of ${R}_{0}$, $S$, ${R}_{d}$ and ${P}_{r}$ on the SFC and Nusselt number for suction and injection case for $\beta =0.2, m=1, Ha=1, {\phi }_{1}={\phi }_{2}={\phi }_{3}=0.02$.

${R}_{0}$	$S$	${R}_{d}$	${P}_{r}$	${C}_{f-1}$ for suction case (${R}_{e}=-0.5$)	${N}_{u} \left |_{\eta =-1}\right.$ for suction case $({R}_{e}=-0.5)$	${C}_{f-1}$ for injection case $({R}_{e}=0.5)$	${N}_{u} \left | _{\eta =-1}\right.$ for injection case $({R}_{e}=0.5)$
1	3	1	6.2	26.6857	1.3614	26.8082	4.0747
2				25.9166	1.3468	26.0394	4.1195
3				25.3150	1.3328	25.4620	4.630
4				4.8833	1.3195	25.0781	4.2049
1	3			26.6857	1.3614	26.8082	4.0747
	5.7			26.6857	1.9644	26.8082	5.2233
	16.2			26.6857	6.0947	26.8082	10.7574
	3	0.5		26.6857	0.9159	26.8082	4.2735
		1		26.6857	1.3614	26.8082	4.0747
		1.5		26.6857	1.8025	26.8082	4.2096
		2		26.6857	2.2419	26.8082	4.4733
			4.5	26.6857	1.4057	26.8082	3.2059
			5.5	26.6857	1.3795	26.8082	3.7016
			6.2	26.6857	1.3614	26.8082	4.0747

**Table 5:** The impact of ${R}_{e}$, $\beta$, Ha, ${\upphi }_{1}$,${\upphi }_{2}$, and ${\upphi }_{3}$ on the SFC and Nusselt number for contraction and expansion case, for ${R}_{0}=1, S=3, {R}_{d}=1, {N}_{1}=1.$

${R}_{e}$	$\beta$	Ha	${\phi }_{1}={\phi }_{2}={\phi }_{3}$	${C}_{f-1}$ for contraction case $(\alpha =-0.5)$	${N}_{u} \left |_{\eta =-1}\right.$ for contraction case $(\alpha =-0.5)$	${C}_{f-1}$ for expansion case $(\alpha =0.5)$	${N}_{u} \left |_{\eta =-1}\right.$ for expansion case $(\alpha =0.5)$
− 2	0.2	1	2%	6.6282	0.1502	6.3339	0.0390
− 0.5				26.6857	1.3614	25.4620	0.4405
0.5				26.8082	4.0747	25.5529	1.7113
2				6.7513	11.2320	6.4257	6.9557
− 0.5	0.2			26.6857	1.3614	25.4620	0.4405
	0.4			15.716	1.3505	14.5098	0.4361
	0.7			11.0645	1.3398	9.8495	0.4316
	1			9.2097	1.3328	8.0018	0.4286
		2		26.6029	1.3587	25.3802	0.4397
		4		26.4289	1.3537	25.2070	0.4380
		6		26.2456	1.3488	25.0236	0.4365
− 0.5	0.2	1	3%	23.0584	1.4021	21.9994	0.4669
			4%	19.8505	1.4434	18.9455	0.4945
			5%	17.0228	1.4854	16.2587	0.5232
			6%	14.5389	1.5279	13.9013	0.5531

**Table 6:** The impact of ${R}_{0}$, $S$, ${R}_{d}$, and ${P}_{r}$ on the SFC and Nusselt number for contraction and expansion case, for $\beta =0.2, m=1, Ha=1, {\phi }_{1}={\phi }_{2}={\phi }_{3}=0.02$.

${R}_{0}$	$S$	${R}_{d}$	${P}_{r}$	${C}_{f-1}$ for contraction case $(\alpha =-0.5)$	${N}_{u}\left |_{\eta =-1}\right.$ for contraction case $(\alpha =-0.5)$	${C}_{f-1}$ for expansion case $(\alpha =0.5)$	${N}_{u} \left |_{\eta =-1}\right.$ for expansion case $(\alpha =0.5)$
1	3	1	6.2	26.6857	1.3614	25.4620	0.4405
2				25.9166	1.3468	24.7154	0.4357
3				25.3150	1.3328	24.1511	0.4312
4				4.8833	1.3195	23.7715	0.4270
1	3			26.6857	1.3614	25.4620	0.4405
	5.7			26.6857	1.9644	25.4620	0.7376
	16.2			26.6857	6.0947	25.4620	3.6209
	3	0.5		26.6857	0.9159	25.4620	0.1564
		1		26.6857	1.3614	25.4620	0.4405
		1.5		26.6857	1.8025	25.4620	0.7897
		2		26.6857	2.2419	25.4620	1.1707
			4.5	26.6857	1.4057	25.4620	0.6329
			5.5	26.6857	1.3795	25.4620	0.5122
			6.2	26.6857	1.3614	25.4620	0.4405

Table 7 provides the comparative study of tri-hybrid nanofluid, hybrid nanofluid, and nanofluid, under the effect of $\alpha$, $\beta$, Ha, and volume fraction on Nusselt number for suction and injection case. For suction case ${R}_{e}<0,$ it is observed that as the value of $\alpha$ moves from negative to positive, the Nusselt number decreases. The increment in $\beta$ decreases the Nusselt number. The influence of Ha and $\beta$ have the opposite effect on the Nusselt number. The larger the value of the volume fraction resulted in the increment in the Nusselt number. For injection case ${R}_{e}>0$, the opposite behavior of the influence of $\beta$, Ha on the Nusselt number is observed and $\alpha ,$ volume fraction has the same effect on the Nusselt number as compared to the suction case. In both cases, it is observed that the Nusselt number or heat transfer rate of tri-hybrid nanofluid is better as compared to hybrid nanofluid and nanofluid flow. Furthermore, the heat transfer rate of hybrid nanofluid flow is better compared to nanofluid flow.

**Table 7:** Comparison of tri-hybrid nanofluid, hybrid nanofluid, and nanofluid.

$\alpha$	$\beta$	Ha	Tri-hybrid nanofluid $\text{Fe}_{3}{\text{O}}_{4}{-}\text{Al}_{2}{\text{O}}_{3}{-}\text{TiO}_{2}/{\text{H}}_{2}\text{O}$			Hybrid nanofluid $\text{Fe}_{3}{\text{O}}_{4}{-}\text{TiO}_{2}/{\text{H}}_{2}\text{O}$		Nanofluid $\text{TiO}_{2}/{\text{H}}_{2}\text{O}$	
			The volume fraction of particles	${N}_{u} \left |_{\eta =-1}\right.$ for injection case	${N}_{u} \left |_{\eta =-1}\right.$ for suction case	${N}_{u} \left |_{\eta =-1}\right.$ for injection case	${N}_{u} \left |_{\eta =-1}\right.$ for suction case	${N}_{u} \left |_{\eta =-1}\right.$ for injection case	${N}_{u} \left |_{\eta =-1}\right.$ for suction case
− 2	0.2	1	2%	9.9240	5.0465	9.0900	4.5755	9.0775	4.5533
− 0.5				4.0747	1.3614	3.6684	1.1646	3.6389	1.1313
0.5				1.7113	0.4405	1.4825	0.3526	1.4472	0.3331
2				0.3234	0.0645	0.2543	0.0459	0.2382	0.0413
− 0.5	0.2			4.0747	1.3614	3.6684	1.1646	3.6389	1.1313
	0.4			4.1105	1.3505	3.7027	1.1548	3.6734	1.1216
	0.7			4.1470	1.3398	3.7377	1.1450	3.7084	1.1120
	0.2	1		4.1716	1.3328	3.6684	1.1646	3.6389	1.1313
		2		4.0829	1.3587	3.6766	1.1621	3.6479	1.1287
		4		4.0789	1.3597	3.6925	1.1573	3.6652	1.1235
		6		4.1141	1.3488	3.7076	1.1527	3.6817	1.1186
− 0.5	0.2	1	3%	4.1035	1.4021	3.7022	1.1985	3.6787	1.1643
			4%	4.1288	1.4434	3.7353	1.2330	3.71980	1.1979
			5%	4.1537	1.4854	3.7678	1.2683	3.7569	1.2321
			6%	4.1774	1.5279	3.7998	1.3043	3.7954	1.2670

For purpose of results validation, Table 8 displays a comparison of numerical values of skin friction $f{^{\prime}}{^{\prime}}(0)$ against magnetic parameter with that of Zubair et al.^60^ in the special case when $k=0,$ ${\phi }_{1}=0$, ${\phi }_{2}=0.1, {\phi }_{3}=0$, $m=0,$ and $\beta \to \infty$. We note from the table that the present results have excellent agreement with the results of the literature^60^.

**Table 8:** Comparison of $f{^{\prime}}{^{\prime}}(-1)$ against magnetic parameter when $k=0,$ ${\phi }_{1}=0$, ${\phi }_{2}=0.1, {\phi }_{3}=0$, $m=0,$ and $\beta \to \infty$.

$Ha$	Present values	Ref.^60^
0	1.164127	1.1640
2	1.461731	1.4616
4	1.732458	1.7323
6	1.981993	1.9819

## Conclusion

In this article, we examined the magnetohydrodynamic flow and heat transfer of micropolar tri-hybrid nanoparticles between two surfaces. Both surfaces are assumed permeable and move up or down with the rate of ${a}^{{\prime}}(t)$. The modeled problem is transformed into a set of nonlinear ODEs. MATLAB-based Bvp4c method is used to solve this nonlinear system of ODE. As a tri-hybrid substance is a material that combines both physical and chemical characteristics of different materials and then provides these properties in an upgraded and standardized form, this model can be applied in the area of heat transmission such as transportation, and medical sciences. The effect of various dynamic parameters on velocity, micropolar velocity, rotational velocity, ad temperature profiles has been explored in this work. After a detailed study of the article, it is observed that:

- heat transfer rate of tri-hybrid nanofluid is appreciably better as compared to hybrid nanofluid and nanofluid.
- The Nusselt number increase with increment in volume fraction, shape size factor, and thermal radiation parameter, but decrease with the increase in rotation parameter, $\beta$, $\alpha$ for the suction case.
- The SFC s rise with the increase in magnetic parameter, and reduced with growing values of $\beta$, $\alpha$, ${R}_{0}$.
- The rotational velocity profile increases with the increment in magnetic parameter and decreases with the increment in rotational parameter.
- The increasing behavior is also noticed in micro rotational velocity for augmented values of ${R}_{0}$, $Ha$, and $\beta$.

## Data Availability

All data generated or analyzed during this study are included in this published article.

## List of symbols

$$A$$: Permeability component
${\beta }_{0}$: The strength magnetic field
$${\sigma }_{e}$$: Electrical conductivity
${k}_{nf}$: Thermals conductivity of the nanofluid
${k}_{hnf}$: Thermal conductivity of hybrid nanofluid
${k}_{trihnf}$: Thermal conductivity of tri-hybrid nanofluid
n: Shape factor
${c}_{p}$: Specifics heats at constant pressures
$$Ha$$: Magnetic parameter
${\alpha }_{nf}$: Thermal conductivity for the nanofluid
${\rho }_{nf}$: Density for the nanofluid
${R}_{e}$: Permeability Reynolds numbers
$${P}_{r}$$: Prandtl number
$$\Omega$$: Angular velocity
$${R}_{d}$$: Radiation parameter
${q}_{r}$: Radiative heat flux
$\widehat{J}$: Microinertial per unit mass
SFC: Skin friction coefficients

## Greek symbols

$\alpha$: The wall expansion ratio
$\eta$: Scaled boundary layer coordinate
$$\theta$$: Dimensionless temperature
$$\mu$$: Dynamic viscosity
$$\upsilon$$: Kinematic viscosity
$$\rho$$: Density
$\phi$: Volume fraction of nanoparticles
$$\sigma$$: Electric conductivity

## Subscripts

nf: Nanofluid
hnf: Hybrid nanofluid
trihnf: Tri-hybrid nanofluid
s1: First nanoparticles
s2: Second nanoparticles
s3: Third nanoparticle
